# Unraveling the genetic potential of nitrous oxide reduction in wastewater treatment: insights from metagenome-assembled genomes

**DOI:** 10.1128/aem.02177-23

**Published:** 2024-08-13

**Authors:** Patrick Skov Schacksen, Jeppe Lund Nielsen

**Affiliations:** 1 Department of Chemistry and Bioscience, Aalborg University, Aalborg, Denmark; University of Michigan, Ann Arbor, Michigan, USA

**Keywords:** nitrous oxide reductase, NosZ, nitrous oxide sink, wastewater treatment, nitrogen metabolism

## Abstract

This study explores the genetic landscape of nitrous oxide (N_2_O) reduction in wastewater treatment plants (WWTPs) by profiling 1,083 high-quality metagenome-assembled genomes (HQ MAGs) from 23 Danish full-scale WWTPs. The focus is on the distribution and diversity of nitrous oxide reductase (*nosZ*) genes and their association with other nitrogen metabolism pathways. A custom pipeline for clade-specific *nosZ* gene identification with higher sensitivity revealed 503 *nosZ* sequences in 489 of these HQ MAGs, outperforming existing Kyoto Encyclopedia of Genes and Genomes (KEGG) module-based methods. Notably, 48.7% of the total 1,083 HQ MAGs harbored *nosZ* genes, with clade II being predominant, accounting for 93.7% of these genes. Taxonomic profiling highlighted the prevalence of *nosZ*-containing taxa within *Bacteroidota* and *Pseudomonadota. Chloroflexota* exhibited unexpected affiliations with both the *sec* and *tat* secretory pathways, and all were found to contain the accessory *nosB* gene, underscoring the importance of investigating the secretory pathway. The majority of non-denitrifying N_2_O reducers were found within *Bacteroidota* and *Chloroflexota*. Additionally, HQ MAGs with genes for dissimilatory nitrate reduction to ammonium and assimilatory nitrate reduction frequently co-occurred with the *nosZ* gene. Traditional primers targeting *nosZ* often focus on short-length amplicons. Therefore, we introduced custom-designed primer sets targeting near-full-length *nosZ* sequences. These new primers demonstrate efficacy in capturing diverse and well-characterized sequences, providing a valuable tool with higher resolution for future research. In conclusion, this comprehensive analysis enhances our understanding of N_2_O-reducing organisms in WWTPs, highlighting their potential as N_2_O sinks with the potential for optimizing wastewater treatment processes and mitigating greenhouse gas emissions.

**IMPORTANCE:**

This study provides critical insights into the genetic diversity of nitrous oxide reductase (nosZ) genes and the microorganisms harboring them in wastewater treatment plants (WWTPs) by exploring 1,083 high-quality metagenome-assembled genomes (MAGs) from 23 Danish full-scale WWTPs. Despite the pivotal role of nosZ-containing organisms, their diversity remains largely unexplored in WWTPs. Our custom pipeline for detecting nosZ provides near-full-length genes with detailed information on secretory pathways and accessory nos genes. Using these genes as templates, we developed taxonomically diverse clade-specific primers that generate nosZ amplicons for phylogenetic annotation and gene-to-MAG linkage. This approach improves detection and expands the discovery of novel sequences, highlighting the prevalence of non-denitrifying N_2_O reducers and their potential as N_2_O sinks. These findings have the potential to optimize nitrogen removal processes and mitigate greenhouse gas emissions from WWTPs by fully harnessing the capabilities of the microbial communities.

## INTRODUCTION

Nitrous oxide (N_2_O) in the atmosphere has significantly contributed to stratospheric ozone depletion over the last 150 years, increasing by more than 20% since 1750 (1, 2). This increase is pronounced, given that N_2_O possesses a warming potential almost 300 times that of carbon dioxide (CO_2_) (3). This N_2_O emission is further exacerbated by the estimated annual increase of 2%, attributing to nearly 80% of the carbon footprint in modern wastewater treatment plants (WWTPs) globally (1, 4). In the context of modern WWTPs, nitrogen removal stands as a cornerstone, playing a pivotal role in reducing nitrogen compounds and thereby mitigating N_2_O emissions (5). However, paradoxically, the nitrogen removal process itself gives rise to a substantial portion of anthropogenic N_2_O emissions, constituting approximately 3% of the total N_2_O emissions (6).

Ammonia-oxidizing bacteria (AOB) and incomplete denitrifiers are identified as the primary N_2_O producers during the nitrification and denitrification process. Additionally, other nitrogen-related metabolisms influenced by environmental factors, such as fluctuating levels of ammonia (NH_3_), hydroxylamine (NH_2_OH), or nitrite (NO_2_^−^), have also been associated with N_2_O formation (6, 7). Notably, bacteria performing dissimilatory nitrate reduction to ammonium (DNRA), where nitrate (NO_3_^−^) and NO_2_^−^ are reduced to ammonium (NH_4_^+^), also contribute to N_2_O formation. This process is primarily catalyzed by either the periplasmic cytochrome c nitrate reductase (*nrfA*) or the soluble siroheme-containing enzyme nitrite reductase (*nirB*). The amount of N_2_O released by DNRA-performing bacteria is uncertain but is considered to constitute only a marginal fraction of the overall N_2_O emissions compared to denitrification and AOB activities (8–11).

During the complete denitrification process, NO_3_^−^ and NO_2_^−^ are reduced to gaseous dinitrogen (N_2_) (10, 12). However, since the reduction of NO_3_^−^ to NO_2_^−^ is not exclusive to denitrification, the genes responsible for catalyzing this reaction (*nap*/*nar*) are not considered strictly denitrifying genes. The reduction of N_2_O to N_2_ is catalyzed by N_2_O reductase (*nosZ*) and is the best-known and most well-characterized biological sink for N_2_O in the biosphere (9). Recent studies have also identified another biological sink for N_2_O: N_2_O fixation (N_2_O to NH_4_^+^) in freshwater, linking a subcommunity of nitrogenase (*nifH*)-containing microorganisms to this process (13). The modular denitrification process can be carried out by a single organism reducing NO_3_^−^/NO_2_^−^ to N_2_, referred to as “complete denitrifier,” or by a consortium of specialized cooperating microorganisms known as “partial denitrifiers.” These partial denitrifiers, defined as lacking one or more denitrifying genes, contribute to variations in the net N_2_O emission or consumption, depending on the microbial community’s genetic potential and environmental constraints (9). NosZ-containing non-denitrifying organisms are characterized by the absence of genes required for converting NO_2_^−^ to gaseous nitric oxide (NO) (*nirK*/*S*) and subsequent NO to N_2_O (*norB*/*C*). They offer an alternative pathway for N_2_O reduction, making them essential for understanding and managing N_2_O emissions in WWTPs and other environments (6, 14). The recent evidence of microorganisms directly utilizing N_2_O as a nitrogen source through N_2_O fixation (N_2_O to NH_4_^+^) further highlights the complexity and significance of microbial interactions in N_2_O dynamics (13).

The NosZ protein phylogeny reveals two major clades (clades I and II), each generally containing a unique translocation pathway in the N-terminal of the gene. Most NosZ clade I employ the twin-arginine translocation (*tat*) pathway, transporting the folded protein across the membrane (15, 16). Clade I is further characterized by the presence of the accessory membrane-bound Fe-S flavoprotein gene (*nosR*) found in the *nos* gene cluster (9). The larger NosZ clade II utilizes the secretory pathway (*sec*) at the N-terminal, which facilitates the transport of the unfolded protein across the cytoplasmic membrane (17). Clade II is also distinguished by the presence of a membrane-spanning protein gene (*nosB*) found directly downstream of *nosZ* (9). This protein, in some cases, forms a NosB-NosC2 fusion protein anchored in the membrane by a N-terminal helix (18). Exceptions to these general rules of the secretory pathway and accessory genes do occur, and whether the translocated pathway is related to taxa or clade-specific functional significance is unclear (9). An example of this exemption is the *nosB* gene not being exclusive to clade II with certain exceptions within archaea and specific phyla, e.g., *Chloroflexota* (18). This clade II subgroup of *Chloroflexota* can also harbor the *tat* pathway instead of *sec* (16). The larger clade II *nosZ* group includes most non-denitrifying N_2_O reducers (16, 19). Organisms harboring *nosZ* clade I are most commonly found to also harbor both *norB*/*C* and *nirK*/*S*. While these also occur for clade II, it is more common for clade II to exclude other denitrification-affiliated genes (20). Genetic analysis has shown a distinct clustering of *nosZ* genes to phylogenetic groups and a relatively conserved gene structure, indicating low horizontal gene transfer, and it has, therefore, been hypothesized to hold potential as a reference gene for gene-based phylogenies (21). To date, the majority of primers targeting *nosZ* are based on short-read amplicons, aimed at achieving diverse phylogenetic coverage or targeting specific clades in unspecific environments, sometimes resulting in unspecific primers amplifying a multitude of other genes in the process (19, 22, 23). However, advancements in sequencing techniques, such as Oxford Nanopore, have made it feasible to design full-length, environmentally specific *nosZ* targeting primers. The primary limitation now lies in the specificity and quality of these designed primers (24). Despite the pivotal role of the *nosZ*-containing organisms, the diversity of the *nosZ* gene and the microorganisms harboring it remains largely unexplored in WWTPs. In this study, we investigate the phylogenetic and genetic diversity of *nosZ* in wastewater treatment plants, shedding light on the microorganisms harboring clade-specific *nosZ* genes. Our approach involves the identification of clade-specific *nosZ* genes in a non-targeted metagenomic approach and the design of environmentally specific long-fragment primers based on these genes to address microdiversity. This allows for the potential detection of novel N_2_O-reducing bacteria, as demonstrated in high-diversity full-scale WWTPs, based on high-quality near-full-length *nosZ* genes thereby advancing the detection and understanding of *nosZ-*harboring organisms.

## MATERIALS AND METHODS

### Pipeline for identification, alignment, and phylogeny of clade-specific *nosZ* gene sequences

To identify and annotate *nosZ* genes in activated sludge, 1,083 high-quality metagenome-assembled genomes (HQ MAGs) were obtained from 23 Danish full-scale WWTPs (25). The definition of an HQ MAG is based on the MIMAG draft requirement as >90% completeness; <5% contamination; the presence of 5S, 16S, and 23S rRNA genes; and ≥18 tRNA genes (26). These HQ MAGs were initially processed using Prodigal v2.6.2 to predict protein-coding genes (27). From there, the nucleotide sequences and translated protein sequences were identified and isolated. The identified nucleotide genes were mapped to National Center for Biotechnology Information (NCBI) GenBank v 234 (28) using the BLASTn algorithm, BLAST+ v2.12.0 (29) with a cutoff value of “1*e*^−10^,” and also annotated using KEGG elements (30) utilizing EnrichM v0.5.0 (31) to identify *nosZ* genes. Following the annotation of nucleotide sequences, the translated proteins were mapped using the BLASTp algorithm “1*e*^−10^” to high-quality full-length clade I (*n* = 20) and II (*n* = 46) NosZ protein sequences obtained from the Functional Gene Pipeline and Repository database version v9.9.11 (28). The translated proteins were subsequently mapped using the hmmsearch algorithm from HMMER v3.3.2 (32) to three full-length NosZ Hidden Markov Model files (HMMfiles) [one clade I (638aa) and two clade II (765 and 656aa)] (Text S1). The identified *nosZ* genes were all subset and manually screened for length (1,050–2,200 bp or 350–800aa). The respective taxonomy of the individual HQ MAGs was coupled to the identified *nosZ* genes from which they originated and used throughout.

To obtain additional clade-specific information on the identified NosZ proteins, the secretory pathway was identified through the presence of signal peptides associated with the *sec*/*tat* signal peptides, predicted using PRED-TAT (33). The presence of the clade-specific accessory genes, *nosB* and *nosR*, was verified using BLAST+ v2.12.0 (29). Reference genes for NosB-NosC (*n* = 5) and NosR (*n* = 1) (Text S1) were obtained from previous studies (18, 34), along with a manually curated NosR reference gene database from NCBI (*n* = 64) (Text S1) (35). The identified *nosB* were manually validated to be within seven genes up or downstream of *nosZ* and within three genes up or downstream for *nosR* (29). The *nosZ* genes identified were aligned using MUSCLE v5.0.1428 (36) and subsequently used to construct a maximum likelihood phylogenetic tree using IQ-TREE v2.0 (37), wherein the best-fit model was identified using the IQ-TREE model finder utilizing the minimized Bayesian information criterion score and 1,000 ultrafast bootstrap iterations (38). Consensus among different alignment strategies [MAFFT v7.490 (39) and MUSCLE v5.0.1428 (36)] and tree construction algorithms [maximum likelihood, unweighted pair group method with arithmetic mean (UPGMA), and neighbor joining] ensured systematic grouping of phylogenetic groups (data not shown), with MUSCLE and maximum likelihood trees chosen for their higher protein alignment accuracy (40). The tree was manually curated for misclassified genes.

The constructed trees were visualized using RStudio 2021.09.0+351 (41), R 4.1.2 (42), and the ggtree v3.2.0 package (43) and included taxonomy from the MiDAS 4 full-length v4.8.1 database (44). The HQ MAGs underwent genome-wide taxonomic classification, and their *nosZ* genes were then subset and assigned the taxonomic classification from the HQ MAG they originated from. This subset of taxonomically annotated *nosZ* genes served as a manually curated database throughout the study (30, 31).

### Gene annotation

Using the predicted HQ MAGs translated protein-coding gene sequences from Prodigal v2.6.2 (27), the genes were annotated using EnrichM v0.5.0 (31) and KO annotations (30). A manually curated list of nitrogen metabolism-related KEGG modules was used to investigate the partial and complete KO modules found within the 1,083 HQ MAGs. Pathways were assumed present if 100% of the genes from the investigated list of KEGG modules were present (Table S1). MiDAS 4.8.1 taxonomy and *nosZ* clade-specific information were incorporated into HQ MAG annotations to allow insights into the physiology of relevant *nosZ-*related subgroups.

### *nosZ* clade-specific primer design of taxonomic subgroups

Phylogenetic trees served as a reference to identify and subset distinct subclades based on nucleotide sequences. The subset nucleotide references were processed using CLC Genomic Workbench 20 (45) to generate primer combinations targeting multiple diverse taxonomic genera within the same phylum. Primers were generated based on the following criteria: primer melting temperature (*t*_m_) between 51°C and 58°C, GC content between 40% and 60%, allowed degeneracy of 3, allowed mismatch of 3, and target fragments longer than 1,000 bp. The *nosZ* primers (Table S2) were tested *in silico* against the HQ MAG gene sequences using EMBOSS v6.6.0 (46). The best primer combinations, targeting *nosZ* longer than 1,000 bp with <20% mismatch, and none-to-low ratio of other genes were tested by PCR (data not shown).

### Sampling and DNA extraction

DNA from activated sludge, collected from Aalborg West WWTP (22-01-2020), were extracted using the FastDNA SPIN Kit for Soil (MP Biomedicals, USA). Aalborg West WWTP was chosen as a representative site for the *nosZ* primer test due to its long-term involvement in microbial community studies since 2006 and well-described, high-diversity, and relatively stable microbial community (47). DNA extraction followed the manufacturer’s instructions with minor alterations, using 478 µL sodium phosphate buffer and increased bead beating time to 4 × 40 s on FastPrep FP120 (MP Biomedicals, USA). DNA concentrations were measured with the Qubit dsDNA BR Assay Kit (Life Technologies, USA) on a Tecan Infinite F200 Pro (Tecan, Switzerland), and genomic DNA quality was assessed with a Genomic DNA ScreenTape and TapeStation 2200 (Agilent Technologies, USA).

### Amplification and sequencing of *nosZ* primers

PCR was made in 25-µL reactions using 10 ng template genomic DNA, 0.4 µM forward and reverse primer, and LongAmp Taq Master Mix (New England Biolabs, USA). The thermocycling program used was as follows: initial denaturation at 95°C (3 min), 35 cycles of 95°C denaturation (30 s) followed by gradient annealing depending on the primers used average *T*_m_, *T*_m_ – 5, and *T*_m_ – 10 (60 s), 65°C extension for 100 s, and a final extension at 65°C for 10 min. PCR amplicons were visualized using gel electrophoreses on a ChemiDoc MP Imaging system (Bio-Rad, USA), with a 1% agarose gel (DNA Pure Grade) (Electran, Belgium) and 1× TAE Buffer (AppliChem PanReac, Germany) stained with GelRed (EMD Millipore, USA).

Samples resulting in clear bands with the theoretical target length and no unspecific bands were sequenced using Oxford Nanopore sequencing technologies due to the ability to retrieve full-length *nosZ* amplicons. Sequencing was performed using a MinION Nanopore R9.4.1 flow cell (FLO-MIN106D) with the Ligation Sequencing Kit (SQK-LSK109) and PCR Barcoding kit (EXP-PBC096) (Oxford Nanopore Technologies, UK). The samples were pooled in equimolar concentration according to the manufacturer’s instructions, and the flow cell was run for 14 hours.

### Bioinformatic processing and clade-specific identification of *nosZ* genes

Raw sequenced fast5 Nanopore data were basecalled using guppy v4.2.2 and the dna_r9.4.1_450bps_hac.cfg model from Oxford Nanopore, yielding 0.57 Gbp. Barcodes and adapters were trimmed and demultiplexed using Porechop v0.2.3 (parameters --discard_middle --require_two_barcodes --barcode_threshold 75) (48) and filtered for low-quality reads <80% basecall accuracy (*Q*-score >7) using NanoFilt v2.6.0 (49), keeping only reads between 800 and 2,500 bp. Filtered reads were clustered into operational taxonomic units (OTUs) and denoised using VSEARCH v2.13.4 (parameters --cluster_unoise --minsize 1 --centroids) (50). The amplicons were mapped to a manually curated database with the *nosZ* gene sequences using QIIME2 v2020.6 (51) and VSEARCH v2.13.4 (QIIME feature-classifier classify-consensus-vsearch) consensus taxonomy classifier (50). Unassigned reads were extracted using seqtk v1.3 subseq function and mapped to the GenBank database release 248 (35) using the BLASTn algorithm and an *e*-value cutoff of “1*e*^−10^.”

## RESULTS

### Profiling of nitrogen metabolism genes in WWTP HQ MAGs

**Fig 1:**
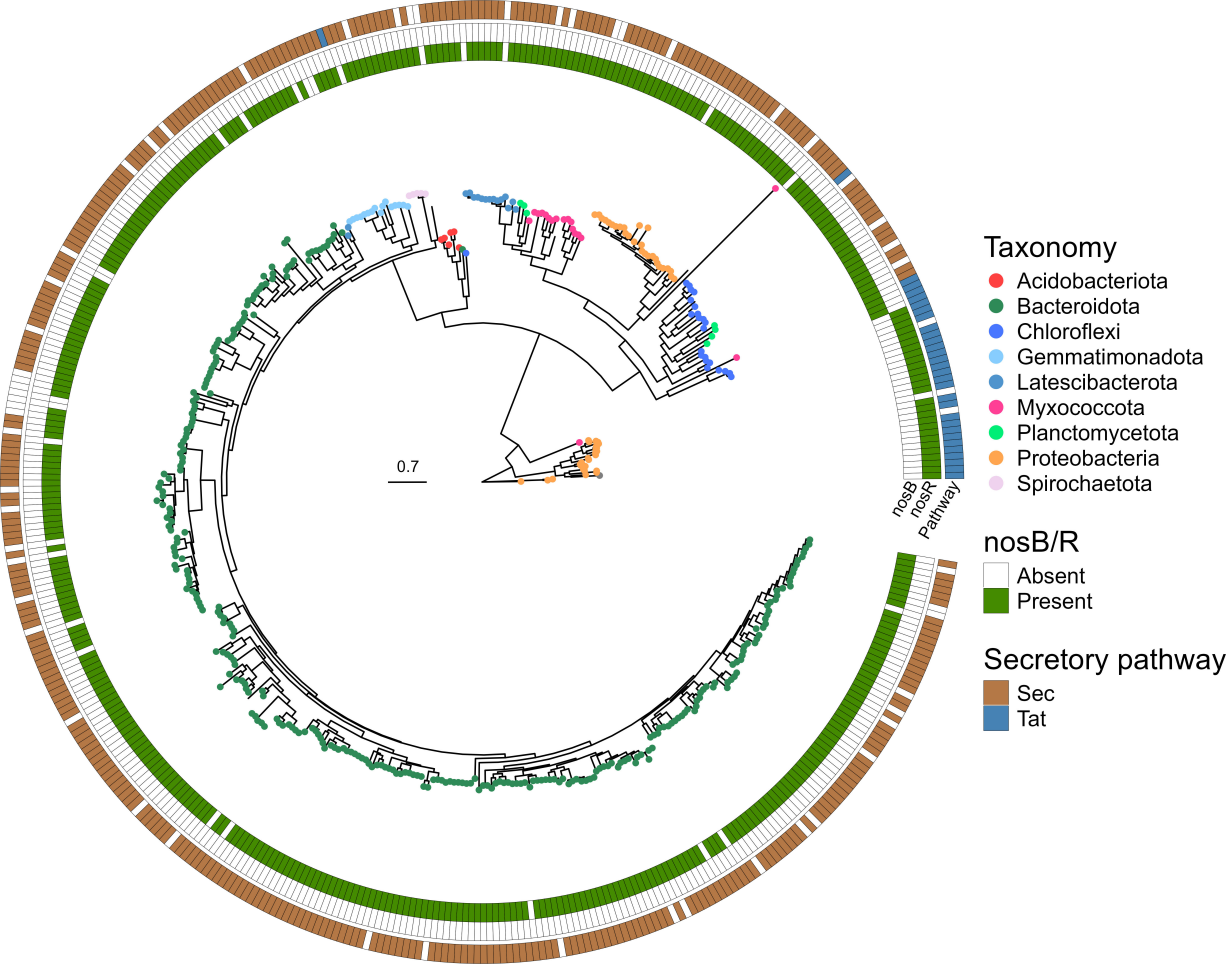
Circular maximum likelihood taxonomic tree of all clade annotated *nosZ* sequences on phylum level longer than 350aa (*n* = 443), identified using the custom pipeline (see Results). The tip of each branch is colored by phylum with the presence/absence of indication of the respective accessory *nosB*/*R* genes and *sec*/*tat* secretory pathway. Ultrafast bootstrap values = 1,000. The scale bar denotes the amino acid substitution rate. The taxonomic classification is made through the MiDAS 4.8.1 database (44).

**Fig 2:**
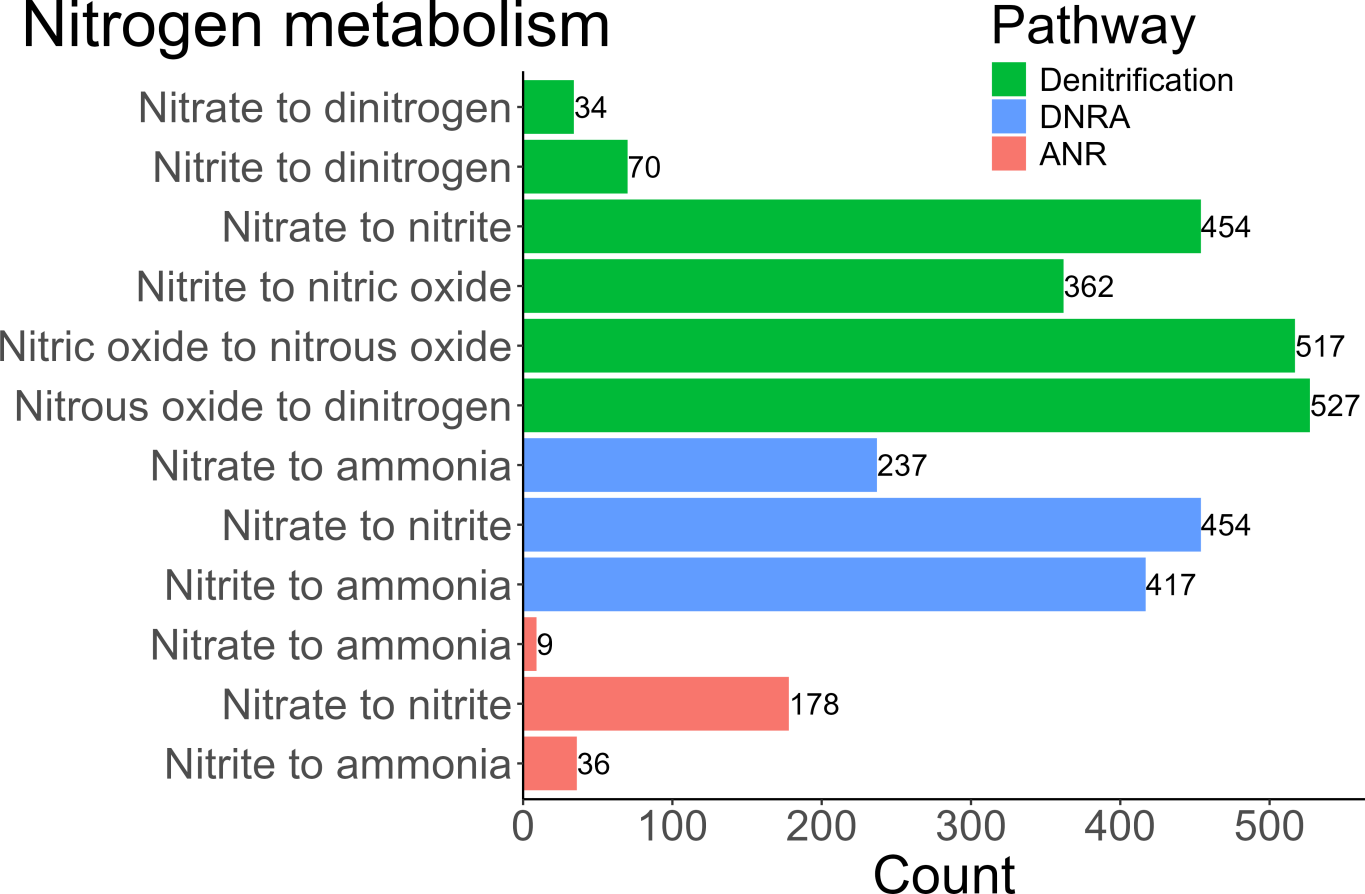
Barplot showing the abundances of nitrogen-related metabolisms and partial pathways in 1,083 HQ MAGs from activated sludge, based on the EnrichM data utilizing the KEGG orthology gene list found in Table S1.

The 1,083 HQ MAGs analyzed in this study originate from a study linking the community structure in activated sludge to their complete genetic repertoires. The HQ MAGs were collected from 23 Danish full-scale WWTPs and were estimated to account for approximately 30% of the relative community abundance, based on 16S rRNA relative community abundance (Fig. S1) (25). Annotating the nitrogen-related metabolisms of the HQ MAGs using KO modules and the EnrichM software revealed the presence of 503 *nosZ* sequences across 489 HQ MAGs. The custom pipeline, designed for clade-specific *nosZ* gene identification, outperformed EnrichM and KO annotations in terms of full-length gene specificity and provided additional information regarding secretory pathways and accessory *nos* genes. It successfully identified 443 unique (cutoff 100% sequence identity) clade-specific *nosZ* genes with a minimum length of 1,050 bp in 428 HQ MAGs (doi.org/10.5061/dryad.p5hqbzkwq), including 38 HQ MAGs missed by EnrichM and the KO annotations (Fig. 1). Among the total 1,083 HQ MAGs, 527 (48.7%) harbored *nosZ* genes. However, only 34 and 70 possessed all the genes necessary for the complete reduction of NO_3_^−^ and NO_2_^−^ to N_2_, respectively. Almost half of all HQ MAGs (517) contained *norB* or *norC,* indicating the potential to reduce NO to N_2_O (Fig. 2). However, among these HQ MAGs containing *norB*/*C*, 216 lacked a *nosZ* gene for further reduction, highlighting incomplete or partial denitrification pathways in over 20% of the 1,083 HQ MAGs.

Phylogenetic analysis of clade-specific *nosZ* genes revealed 27 clade I and 401 clade II *nosZ*-containing HQ MAGs (Fig. 1). From the clade II-containing HQ MAGs, 15 contained two *nosZ* genes, showcasing the diversity of their denitrifying potential within the community. Within the HQ MAGs containing two *nosZ* genes, no two genes were identical nor positioned within 45 genes up or downstream of each other. A complete set of denitrifying genes allowing for the complete reduction of NO_2_^−^ to N_2_ was found in 13 out of the 27 clade I annotated HQ MAGs, with only 9 also containing a gene to reduce NO_3_^−^. Furthermore, among the clade II annotated HQ MAGs, 46 out of 401 HQ MAGs contained the respective genes for fully reducing NO_2_^−^ to N_2_, and 17 of these had the genetic potential to fully reduce NO_3_^−^. Remarkably, 146 HQ MAGs were classified as non-denitrifying N_2_O reducers, defined by the presence of *nosZ* but lacking *norB*/*C* and *nirK*/*S*. Among these, two contained *nosZ* clade I and 144 clade II (Fig. S3 and S4). From these non-denitrifying N_2_O reducers, 21 (13.4%) contained the *nap*/*nar* genes; however, as the reduction of NO_3_^−^ to NO_2_^−^ is not limited to denitrification, these HQ MAGs were also considered non-denitrifying N_2_O reducers.

### Denitrifying and non-denitrifying N_2_O reducers’ connection to DNRA

Of the 1,083 annotated HQ MAGs, 237 contained genes for the complete DNRA pathway, and 9 for complete assimilatory nitrate reduction (ANR) pathway genes. Within these HQ MAGs, genes capable of reducing NO_2_^−^ to NH_4_^+^ were found in 432 HQ MAGs, with 417 via the DNRA pathway and 36 via the ANR pathway. Among these, 21 contained genes from both pathways. Out of the 432 HQ MAGs, 167 also contained *nosZ*, where 91 of these *nosZ*-containing HQ MAGs lacked *norB*/*C*. From the 27 *nosZ* clade I-containing HQ MAGs, 20 (74.1%) contained genes to reduce NO_2_^−^ to NH_4_^+^, with 7 (26.9%) of these lacking *norB*/*C*. From the 401 *nosZ* clade II-containing HQ MAGs, 126 (31.4%) contained genes to reduce NO_2_^−^ to NH_4_^+^, 70 (17.5%) of which lacked *norB*/*C*.

### Taxonomic profiling of *nosZ* clade I-containing HQ MAGs

Phylogenetic diversity among the HQ MAGs identified as belonging to *nosZ* clade I was investigated using the MiDAS v4.8.1 reference database (44). A total of 27 HQ MAGs contained *nosZ* clade I, primarily within *Pseudomonadota* and a single hit of *Myxococcota* (Fig. S5). Within the clade I-containing HQ MAGs, 9 families (2 being taxonomic placeholders) and 12 genera (3 being taxonomic placeholders) were annotated. A list of reports describing denitrification capabilities of genera identified to contain *nosZ* clade I or II from this study is collected in Table S3.

*Pseudomonadota*, identified to contain both clade I and II annotated *nosZ* genes*,* accounted for 26 *nosZ* clade I HQ MAGs. These were divided into multiple subclades with diverse genetic sequences, with 24 out of 26 hits affiliated to the class *Gammaproteobacteria*. The largest family, *Comamonadaceae* (10 HQ MAGs), contained two genera previously affiliated with denitrification, namely, *Ottowia* (two HQ MAGs) and *Rhodoferax* (six HQ MAGs). Within the *Rhodocyclaceae* family (four HQ MAGs), two genera were affiliated with denitrification, *Candidatus* Accumulibacter (one HQ MAG) and *Zoogloea* (three HQ MAGs). From the *Hahellaceae* family (one HQ MAG), *Hahella* (one HQ MAG) was associated with denitrification and DNRA. The less abundant family *Pseudomonadaceae* (one HQ MAG) containing *Pseudomonas* (one HQ MAG) was also a known denitrifying organism. The single other annotated HQ MAGs affiliating within clade I was within *Myxococcota*, previously classified with the same phyla as *Pseudomonadota*, and was only annotated to the placeholder order UASB-TL25.

### Taxonomic profiling of non-denitrifying N_2_O-reducing clade I-containing HQ MAGs

Examining the taxonomic diversity of the clade I non-denitrifying N_2_O reducers revealed two HQ MAGs (Fig. S3 and S4). Both HQ MAGs were affiliated with the phylum *Pseudomonadota* and the family *Comamonadaceae* and were annotated as belonging to the genera *Limnohabitans* and *Rhodoferax*. Additionally, both HQ MAGs contained *nap*/*nar* and *nosR* genes.

### Taxonomic profiling of *nosZ* clade II-containing HQ MAGs

The *nosZ* clade II-containing HQ MAGs exhibited higher diversity at the phylum level compared to clade I, constituting a substantial portion of the data set (401 of 1,083 HQ MAGs) (Fig. S6). Within the clade II-containing HQ MAGs, 15 contained two *nosZ* genes, summing up to 416 *nosZ* clade II genes. These HQ MAGs were affiliated with Bacteroidota (10 HQ MAGs), *Latescibacterota* (2 HQ MAGs), *Acidobacteriota* (1 HQ MAG), and *Chloroflexota* (1 HQ MAG). For simplicity, HQ MAGs containing multiple *nosZ* genes are henceforth referred to as single HQ MAGs unless otherwise specified.

*Bacteroidota* dominated the *nosZ* clade II-containing HQ MAGs, comprising 70.6% of the total, followed by *Pseudomonadota* (6.5%), *Chloroflexota* (5.5%), *Myxococcota* (4.2%), *Gemmatimonadota* (4%), *Latescibacterota* (3.5%), *Planctomycetota* (1.7%), *Acidobacteriota* (1.2%), *Spirochaetota* (1.2%), and six other phyla, each with a single HQ MAG. In total, 66 families and 144 genera were annotated among clade II-containing HQ MAGs, with 37 of the families and 116 genera being placeholders (Table S3). Within the predominant phylum *Bacteroidota* (283 HQ MAGs), the order *Chitinophagales* (154 HQ MAGs) contained the known denitrifying genera *Ferruginibacter* (20 HQ MAGs), *Terrimonas* (18 HQ MAGs), and *Phaeodactylibacter* (2 HQ MAGs). In addition to the known denitrifiers, the genus *Haliscomenobacter* (4 HQ MAGs) has been associated with the presence of *nosZ*. The family *Saprospiraceae* (88 HQ MAGs) has also previously been associated with denitrification. Other orders, like *Sphingobacteriales* (46 HQ MAGs) and *Flavobacteriales* (45 HQ MAGs), also contained known denitrifiers. From the less abundant *Ignavibacteriales* order (10 HQ MAGs), *Ignavibacteriaceae* (4 HQ MAGs) was known to be affiliated with both *nosZ* and the ability to reduce NO_3_^−^ via the DNRA pathway. The 26 clade II annotated *Pseudomonadota* HQ MAGs were predominantly in the class *Gammaproteobacteria* (23 HQ MAGs) and a few in *Alphaproteobacteria* (3 HQ MAGs). Noteworthy genera affiliated with denitrification were found in *Rhodocyclaceae*, with *Dechloromonas* (5 HQ MAGs), *Denitratisoma* (3 HQ MAGs), *Ferribacterium* (1 HQ MAG), and *Sulfuritalea* (5 HQ MAGs). Affiliated with the *Comamonadaceae* family were two other denitrifying genera, namely, *Rhodoferax* (1 HQ MAG) and *Sphaerotilus* (1 HQ MAG). The 22 clade II annotated *Chloroflexota* HQ MAGs were primarily in the *Anaerolineae* class (17 HQ MAGs). Among these, *Candidatus* Amarolinea (2 HQ MAGs) was associated with DNRA and non-denitrifying N_2_O reduction. In the class *Chloroflexia* (1 HQ MAG), *Kouleothrix* was previously associated with denitrifying capabilities. A total of four HQ MAGs were identified to contain the tat pathway generally affiliated with clade I (16). All four HQ MAGs were affiliated to the class OLB14, and only one family placeholder was identified, namely, midas_f_731 with two genera: midas_g_731 (3 HQ MAGs) and midas_g_1314 (1 HQ MAG). *Myxococcota* (17 HQ MAGs) featured a single genus annotation, *Haliangium* (4 HQ MAGs), associated with denitrification within the *Haliangiaceae* family. *Gemmatimonadota* (16 HQ MAGs) belonged to the *Gemmatimonadaceae* family, with one HQ MAG annotated as *Gemmatimonas*, known to be associated with *nosZ. Acidobacteriota* (5 HQ MAGs) showed potential as non-denitrifying N_2_O reducers, particularly within the orders *Thermoanaerobaculales* and *Vicinamibacterales*, known to be associated with *nosZ* and DNRA, but not complete denitrification. All five HQ MAGs affiliated with *Spirochaetota* were identified within the same genus *Leptospira*, which had previously been associated with non-denitrifying N_2_O reduction.

### Taxonomic profiling of non-denitrifying N_2_O-reducing clade II-containing HQ MAGs

Among *nosZ* clade II-containing non-denitrifying N_2_O reducers, 144 HQ MAGs were identified (Fig. S3 and S4). These were predominantly affiliated with *Bacteroidota* (77.1%), followed by *Chloroflexota* (11.1%), *Gemmatimonadota* (2.8%), *Planctomycetota* (2.8%), *Acidobacteriota* (2.1%), *Myxococcota* (1.4%), and four less abundant phyla, each with a single hit. At the genus level, 64 taxonomic placeholders were found, and only 12 nomenclature genus-level annotations. HQ MAGs containing the *nap*/*nar* genes and the DNRA genes for the reduction of NO_2_^−^ to NH_4_^+^ within the clade II non-denitrifying N_2_O reducers were not specifically allocated to a single phylum. Out of the 144 HQ MAGs, 16 contained the *nap*/*nar* genes to reduce NO_3_^−^ to NO_2_^−^, distributed across various phyla.

### Phylogenetic affiliation of amplified *nosZ* genes

**Fig 3:**
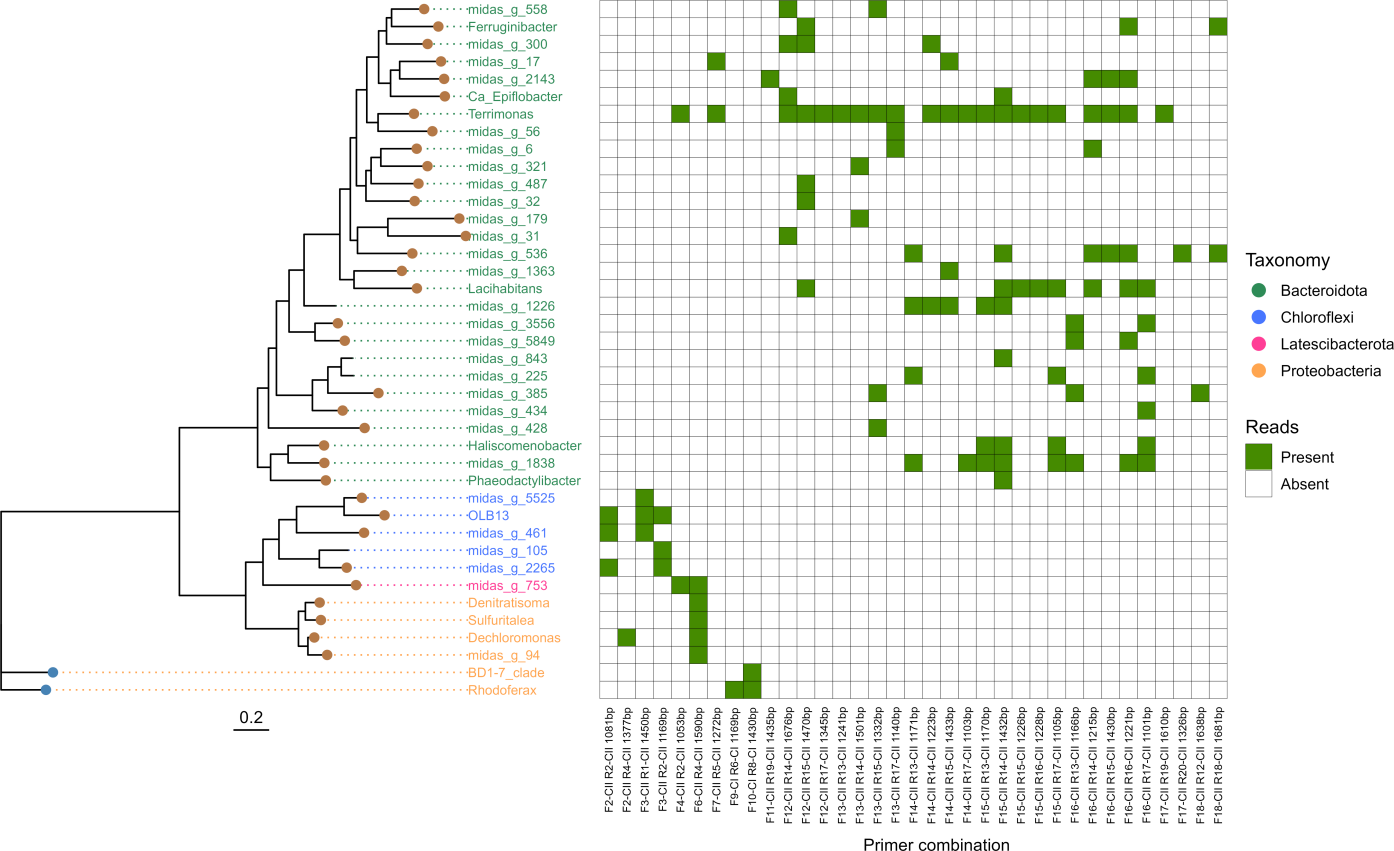
Maximum likelihood taxonomic tree displaying the amplified *nosZ* sequences mapped to the manually curated *nosZ* clade-specific database. The tree is constructed with ultrafast bootstrap values = 1,000 and a scale bar denoting the amino acid substitution rate. Each branch’s tip represents the secretory pathway type, *tat* (blue) and *sec* (brown), of reference genera color-coded according to their respective phyla. Additionally, a joined heatmap illustrates amplicon hits mapped to the reference. The axis names of the heatmap indicate the forward and reverse primers, the nosZ clade they target, and the theoretical length of the amplicon in base pairs (see Table S2 for additional information).

For the investigation of Aalborg West WWTP sludge (selected for its well-characterized status, high microbial diversity, and stable community), 52 primer combinations were systematically assessed. The resulting amplicons yielded 39,592 reads using the custom-designed primers (Table S2). All tested primer combinations produced amplicons with the expected lengths, and 35 resulted in sequences mapped to the manually curated *nosZ* clade-specific database (Fig. 3). Utilizing the custom-designed clade-specific *nosZ*-targeting primers and the 443 identified *nosZ* genes as a reference database, 268 *nosZ* clade I reads and 24,136 clade II reads were taxonomically annotated. The effectiveness of these primers exhibited substantial variability, with a pronounced preference for *nosZ* clade II over clade I, with combinations targeting *Bacteroidota* yielding the highest number of OTUs. From the clade I-targeting primers, two primer combinations produced reads mapped to two distinct clade-specific genera: specifically *Rhodoferax*, a recognized denitrifying genus, and the placeholder BD1-7_clade, described at the family level of *Spongiibacteraceae*. Unfortunately, the development of universal primer combinations capable of amplifying a broader taxonomic diversity beyond one to two phyla and a maximum of five annotated genera proved unattainable. Notably, two primer combinations, F4-CII - R2-CII (designed for targeting *Latescibacterota*) and F7-CII - R5-CII (*Pseudomonadota*) exhibited cross-reactivity with *Bacteroidota*, targeting the genus *Terrimonas*. Additionally, the primer combination F6-CII - R4-CII (*Pseudomonadota*) unintentionally targeted the placeholder genus midas_g_753 within *Latescibacterota*. The *nosZ* clade II-targeting primers exhibited versatility, hitting 38 different genera to varying degrees, encompassing known denitrifiers such as *Dechloromonas*, *Denitratisoma*, *Sulfuritalea*, *Terrimonas*, and *Phaeodactylibacter*. Other genera, including *Haliscomenobacter* and *Ferruginibacter*, known for their affiliation with *nosZ* and varying denitrification abilities, were identified. Two genera, *Lacihabitans* and *Candidatus* Epiflobacter, previously unaffiliated with denitrification or *nosZ*, were also hit. The remaining 29 genera were categorized as MiDAS placeholders. The prominent amplicons, as indicated by the number of primer hits from clade II, were annotated as *Haliscomenobacter*, *Lacihabitans,* and *Terrimonas*, commonly found in Danish WWTPs (44). Among the clade II targeting primer combinations, seven resulted in a single genus-level annotation, while the remaining combinations hit between two and nine genera annotations per combination (Fig. 3).

Following mapping to the manually curated clade-specific *nosZ* database, unassigned reads underwent further analysis using BLASTn (*e*-value 1*e*^−10^), mapping to the NT GenBank v248 database. The subsequent mapping revealed that 5,148 unassigned reads were mapped to various genes, with 2,566 mapped to partial or full-length *nosZ* genes. This mapping encompassed 37 of the evaluated primer combinations.

## DISCUSSION

In this study, a comprehensive analysis of 1,083 HQ MAGs from 23 Danish WWTPs (25) was conducted to identify clade-specific full-length genes of *nosZ* and their taxonomic distribution, utilizing the MiDAS 4.8.1 database (44). The data have been reported to represent around 30% of the microbial community, with almost half (515) of the organisms observed in at least one sample with a relative read abundance above >0.1% of the total population (25). This data set represents the most complete HQ MAG database from activated sludge published to date and provides valuable insight into the most common organisms in modern WWTPs.

### Phylogeny of nosZ

The observation that 48.7% of all investigated HQ MAGs contain at least one copy of the *nosZ* gene highlights the prevalence, distribution, and potentially essential role of nitrous oxide-reducing bacteria within WWTPs. Notably, the distribution of clade I (6.3%) and clade II (93.7%) *nosZ* resembles the tendencies reported in previous studies from soil (9, 52). Further investigation into HQ MAGs containing multiple *nosZ* genes revealed heterogeneous genetic sequences, indicating potential genetic diversity within *nosZ*, even within the same species. The purpose of multiple heterogenous *nosZ* genes remains unclear, as does their origin, which could result from horizontal gene transfer, mutations, gene duplication, diversification, or other mechanisms (21, 53). The phylogenetic relationship of *nosZ* clades, their respective translocation pathway, and the accessory *nos* gene cluster has been extensively studied (9, 54). Despite the typical association of the *tat* pathway and accessory *nosR* gene with clade I, and the *sec* pathway with the accessory *nosB* gene with clade II, exceptions have been reported, particularly within archaea or *Chloroflexota* (9). The structural alignment, shown in Fig. S2, indicates that some *Chloroflexota* contain the *tat* pathway, despite their relation to clade II. This suggests a complex interplay between *nosZ* clades, translocation pathways, and accessory gene clusters, highlighting the need for further research to elucidate the mechanisms driving *nosZ* diversity and function in microbial communities.

### Taxonomic profiling of clade I and clade II

The taxonomic profiling of *nosZ* found in activated sludge revealed a diverse microbial community predominantly affiliated with *Bacteroidota* (66.1%), followed by *Pseudomonadota* (12.2%), *Chloroflexota* (5.1%), *Myxococcota* (4.2%), *Gemmatimonadota* (3.7%), *Latescibacterota* (3.3%), and less abundant phyla (5.4%). These findings align with the typical microbial composition observed in Danish WWTPs (9, 16). This implies a consistent preference of the *nosZ*-containing community across different environments, likely reflecting the natural distribution of the gene within the core microbial community of the specific environment (47, 55). Moreover, it could suggest a widespread energetic advantage associated with having an active *nosZ* enzyme. The largest *nosZ*-containing taxonomic group, *Bacteroidota*, was affiliated with clade II, consistent with previous observations (9). Despite their classification as *nosZ* clade II denitrifiers, many of these HQ MAGs also carried other denitrifying genes, such as *norB*/*C* and *nirK*/*S*. This diversity suggests the potential for partial denitrification or non-denitrifying N_2_O reduction, indicating the key role of these organisms in activated sludge as potential N_2_O sinks (16, 54). Phylogenetic analysis revealed genetic variation within *Pseudomonadota*, with affiliations to both *nosZ* clade I and clade II. The different clades displayed a clear association between the genetic structure, the *tat*/*sec* translocation pathway, and surrounding *nosB*/*R* genes, with both exhibiting genes typical of denitrification. This underscores their primary related role as complete denitrifiers, further supported by the numerous described genera within clade I and *Pseudomonadota* in general. In contrast, *Chloroflexota* did not adhere to the typical clade-specific trend with secretory pathway and accessory genes. Analysis of the phylogenetic relationship (Fig. S2) revealed that the *nosZ* genes within the phylum *Chloroflexota* exhibited a genetic structure similar to other clade II genes. However, four genes (highlighted in green in Fig. S2) containing the *tat* pathway, commonly associated with clade I, also contained the accessory *nosB* gene generally associated with clade II. Consequently, the phylum does not conform to the typical clade-associated trends for *nosZ*. Despite this, all identified *Chloroflexota* in this study were annotated as clade II based on structural alignment. The study emphasizes the importance of investigating the *sec*/*tat* secretory pathway, accessory *nos* genes, and structural alignment to other *nosZ* genes for accurate classification (9, 16, 56). Further exploration of denitrifying genes within the HQ MAGs annotated as *Chloroflexota* revealed that no HQ MAG contained *norB*/*C* genes (Fig. S2) and that approximately 25% contained *nirK* or DNRA/ANR genes, suggesting a denitrifying repertoire more akin to clade II than clade I, regardless of the *sec*/*tat* pathway. The HQ MAGs identified within the largely undescribed *Latescibacterota* phylum contained *nosZ* clade II, highlighting specialized nitrogen-related metabolic potentials for the reduction of NO and N_2_O, and the genes for NO_2_^−^ reduction to NH_4_^+^ via DNRA. Notably, none of the HQ MAGs contained *nirK*/*S*, emphasizing a unique metabolic profile of this phylum. Overall, the high abundance of organisms harboring *nosZ*, particularly those lacking the preceding *norB*/*C* genes for the conventional denitrifying pathway, may indicate multiple functions. This potential function may encompass the utilization of excess or freely available N_2_O to access an additional energy source, thus serving as a beneficial gene for net energy conservation or acting as an electron sink. Additionally, *nosZ* might facilitate the conversion of residual N_2_O from bacteria performing reduction of NO_2_^−^ to NH_4_^+^ via DNRA or ANR (8, 57, 58).

### Distribution of the *nosZ* gene and other related metabolic pathways in the HQ MAGs

The distribution of clade-specific *nosZ* genes within the 1,083 HQ MAGs exhibits a similar distribution with *nosZ* clade II (93.7%) displaying greater diversity compared to clade I (6.3%) as previously described (9). Although this distribution is consistent, a more detailed taxonomic classification of the organisms harboring *nosZ* clade I was observed within this data set. This could result from cultivation biases associated with the species containing *nosZ* when first discovered (16, 59). Despite its larger and more diverse nature, *nosZ* clade II contains fewer described organisms at the genus level. This extensive novelty and number of unique *nosZ* clade II genes demonstrate substantial novelty within this group, suggesting the presence of numerous species still to be characterized.

The metabolic capabilities related to the clade-specific *nosZ* genes align with previous reports, indicating that clade I is more commonly associated with denitrification (39.4% clade I and 11.6% clade II complete denitrifiers) and other nitrogen-related pathways, such as DNRA and ANR (54.5% DNRA, 27.3% ANR clade I), compared to clade II (31.6% DNR, 5.8% ANR) (60). Results from this study show co-occurrence of DNRA genes found among non-denitrifying N_2_O reducers, with 49 of the 146 reducers harboring DNRA genes and only 8 possessing ANR genes. DNRA and ANR pathways, in contrast to denitrification, are undesired in WWTPs as they convert NO_2_^−^ back to NH_4_^+^ rather than removing it from the system as gaseous N_2_ (61). The partial denitrifiers containing *nirK*/*S* but lacking *norB*/*C* constituted 8 of the 27 HQ MAGs affiliating with *nosZ* clade I and only 40 of the 401 with *nosZ* clade II, further strengthening the observation that *nosZ* clade I is more commonly associated with denitrification than clade II, thus true for partial denitrifiers as previously postulated (19).

### Custom-designed *nosZ* targeting primer

The custom-designed primer sets, strategically crafted to target specific near-full-length genetically or taxonomically similar *nosZ* subclades, allow for the enhanced detection and characterization of known environmentally specific *nosZ* genes in WWTPs. While multiple studies have focused on *nosZ* targeting primers and their subclades, the vast majority have concentrated on short-read targeting, primarily due to previous restrictions with sequencing technologies (19, 22, 23). While developing universal primers for near-full-length amplification of *nosZ* was not possible using this strategy, the focus was placed on targeting environmentally diverse groups spanning multiple taxa to address the microdiversity of the different *nosZ* clades. The variable regions of the *nosZ* gene across these clades allow for distinct taxonomic identification, which makes the designed primers valuable candidates for supplementing previously published primers for more comprehensive investigations into a wider selection of *nosZ* genes (19, 21). For clade I-targeting primers, HQ MAGs identified as *Pseudomonadota* were selected as reference clades due to their taxonomic and genetic sequence similarity, containing semi-conserved regions. Concerning clade II-targeting primers, *Bacteroidota* emerged as the primary target, suggesting that the primer designed to target *Acidobacteriota* and *Gemmatimonadota* did not effectively target the intended phyla. A closer examination of HQ MAGs originating from Aalborg West WWTP revealed the expected presence of both phyla in the sludge. It, therefore, appears that the primers targeting phyla other than *Bacteroidota* had lower specificity to the targeted microorganisms or were present in low concentrations in the sample. Notably, from the *nosZ* clade II-targeting primers, seven combinations hit more than five different genera, demonstrating promising potential as *Bacteroidota*-targeting primers. The near-full-length amplicons produced with the newly designed primers revealed a large number of uncharacterized microdiversity within denitrification *nosZ*. These designed primers were also all demonstrated to exhibit high specificity to *nosZ*, providing superior sequence information compared to previous primers with shorter amplicon lengths (62). An optimization strategy for the primers entails considering multiplexing, as various primer combinations have shown the ability to amplify beyond the target genes across different genera and even phyla. Multiplexing primers known to amplify multiple genera could enhance the detection of organisms in a single run, thereby broadening the scope of the analysis. Further validation of primer specificity could involve incorporating additional samples from other WWTPs. However, the primary focus of designing and applying the new primers was to validate taxonomically specific full-length nosZ amplification, as evidenced by both *in silico* and *in vitro* tests. This validation encompassed some of the primary taxonomic targets, known to be prevalent genera in Danish WWTPs; hence, no additional plants were included in the *in vitro* test (44).

### The N_2_O sink potential of the investigated HQ MAGs in activated sludge

The investigated HQ MAGs in this study highlight the potential of both described and undescribed organisms as N_2_O sinks, encompassing both clade I and II-containing organisms (56). Theoretically, these organisms could be harnessed by favoring their presence and/or activity. However, the majority of these relatively abundant organisms in WWTPs remain largely undescribed, with the majority of their genetic repositories unknown (44). From this study, the population of the non-denitrifying N_2_O reducers constitutes around 13.5% of the investigated community, suggesting that a significant portion of the WWTP community has the potential to act as a N_2_O sink and limit emissions, with especially the clade II-containing *Chloroflexota* and *Bacteroidota* emerge as abundant populations. It is important to note that the total 1,083 HQ MAGs analyzed were previously estimated to represent 30% of the activated sludge population (25). Due to the diverse representatives and frequent occurrence of these organisms in Danish WWTPs, a comprehensive exploration of their complete genetic repositories is warranted. Such investigations hold the potential to enhance our understanding of how these organisms may help reduce the carbon footprint by lowering greenhouse gas emissions from WWTPs. Notably, the developed primers offer the opportunity to target taxonomically specific organisms of interest and, thus, assist in specialized analysis of *nosZ* presence or distribution. This knowledge could be invaluable in designing pilot studies aimed at promoting the presence and/or activity of these organisms. The utilization of these organisms as potential N_2_O sinks aligns with the broader goal of optimizing wastewater treatment processes and mitigating greenhouse gas emissions. Further research into the genetic makeup and influencing factors associated with these HQ MAGs is crucial for advancing our strategies in wastewater management. This includes a broader examination of other metabolic pathways and general functions found within them. Such comprehensive analysis could serve as the foundation for targeted studies aimed at investigating various organisms or metabolic functions of interest.

### Conclusion

In this study, we developed a robust pipeline utilizing 1,083 previously published HQ MAGs obtained from 23 full-scale WWTPs in Denmark to identify near-full-length *nosZ* sequences. This approach provided an in-depth analysis of the genetic repertoire of *nosZ*-containing bacteria within wastewater treatment systems.

Our results showed that nearly 48% of the HQ MAGs contained a *nosZ* gene, demonstrating its widespread distribution across various taxa. Additionally, we observed a high co-occurrence of bacteria containing genes associated with DNRA and *nosZ*. HQ MAGs featuring clade I *nosZ* gene exhibited a notable association with other denitrifying genes and complete denitrification, whereas clade II contained a majority of partial denitrifiers and a much lower abundance of complete denitrifiers. There was also a strong association between the secretory pathway and accessory *nos* genes to clade, with *tat* and *nosR* linked to clade I, and *sec* and *nosB* to clade II, except for a subgroup of *Chloroflexota* containing *tat* and *nosB*. Non-denitrifying N_2_O reducers were primarily found among clade II, with only two clade I-containing HQ MAGs identified as non-denitrifying N_2_O reducers. These findings indicate that the role of *nosZ*, especially among clade II, could serve as an additional energy-producing pathway for a substantial portion of the organisms inhabiting activated sludge.

## Data Availability

Genomic data used in this study have been published previously (25) and were accessed from the National Center for Biotechnology Information (NCBI) BioProject database with the accession number PRJNA629478. The sequence data presented in this study were deposited in the European Nucleotide Archive (ENA) database under accession number PRJEB58728.

## SUPPLEMENTAL MATERIAL

Text S1 aem.02177-23-s0001.txt *nosR* and *nosB* supplemental fasta files.

Supplemental figures aem.02177-23-s0002.pdf Figures S1 to S6.

Table S1 aem.02177-23-s0003.xlsx Taxonomy of all 1,083 HQ MAGs analyzed.

Table S2 aem.02177-23-s0004.docx List of custom-designed, clade-specific *nosZ* primers.

Table S3 aem.02177-23-s0005.docx List of clade-specific *nosZ*-containing genera and their nitrogen-related metabolism.

ASM does not own the copyrights to Supplemental Material that may be linked to, or accessed through, an article. The authors have granted ASM a non-exclusive, world-wide license to publish the Supplemental Material files. Please contact the corresponding author directly for reuse.
