# Supplementary material for: Unraveling the genetic potential of nitrous oxide reduction in wastewater treatment: insights from metagenome-assembled genomes
Source: Appl Environ Microbiol. 2024 Aug 13;90(9):e02177-23. doi: 10.1128/aem.02177-23 (PMC11409646; doi:10.1128/aem.02177-23)
Supplement: Supplemental figures — Figures S1 to S6. [file aem.02177-23-s0002.pdf]

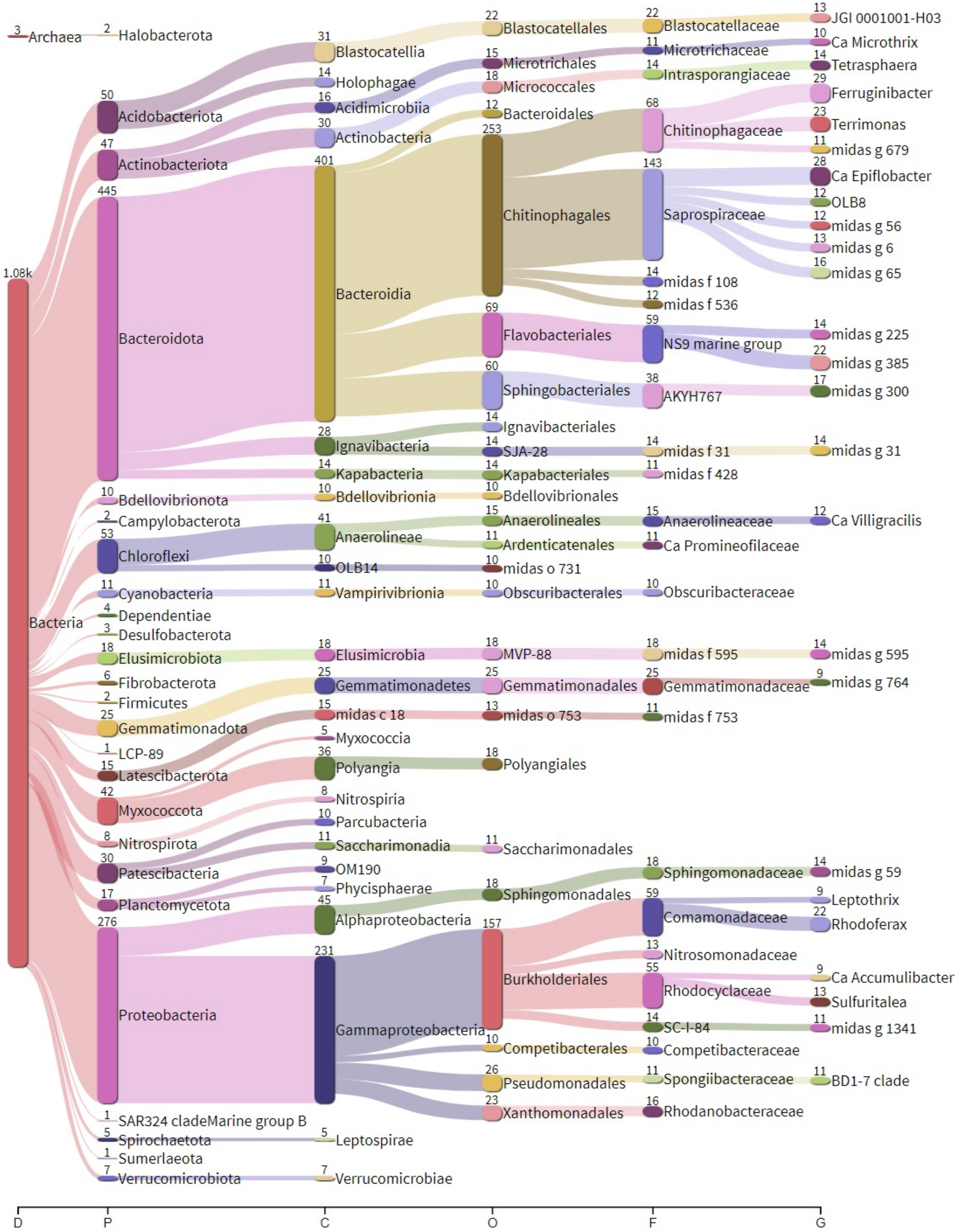

Figure S1: Sankey map of the top 25 taxa shown at each level of the HQ MAGs (n=1083) mapped to the MiDAS v4.8.1 database. Including MiDAS placeholders for annotations without binomial nomenclature.

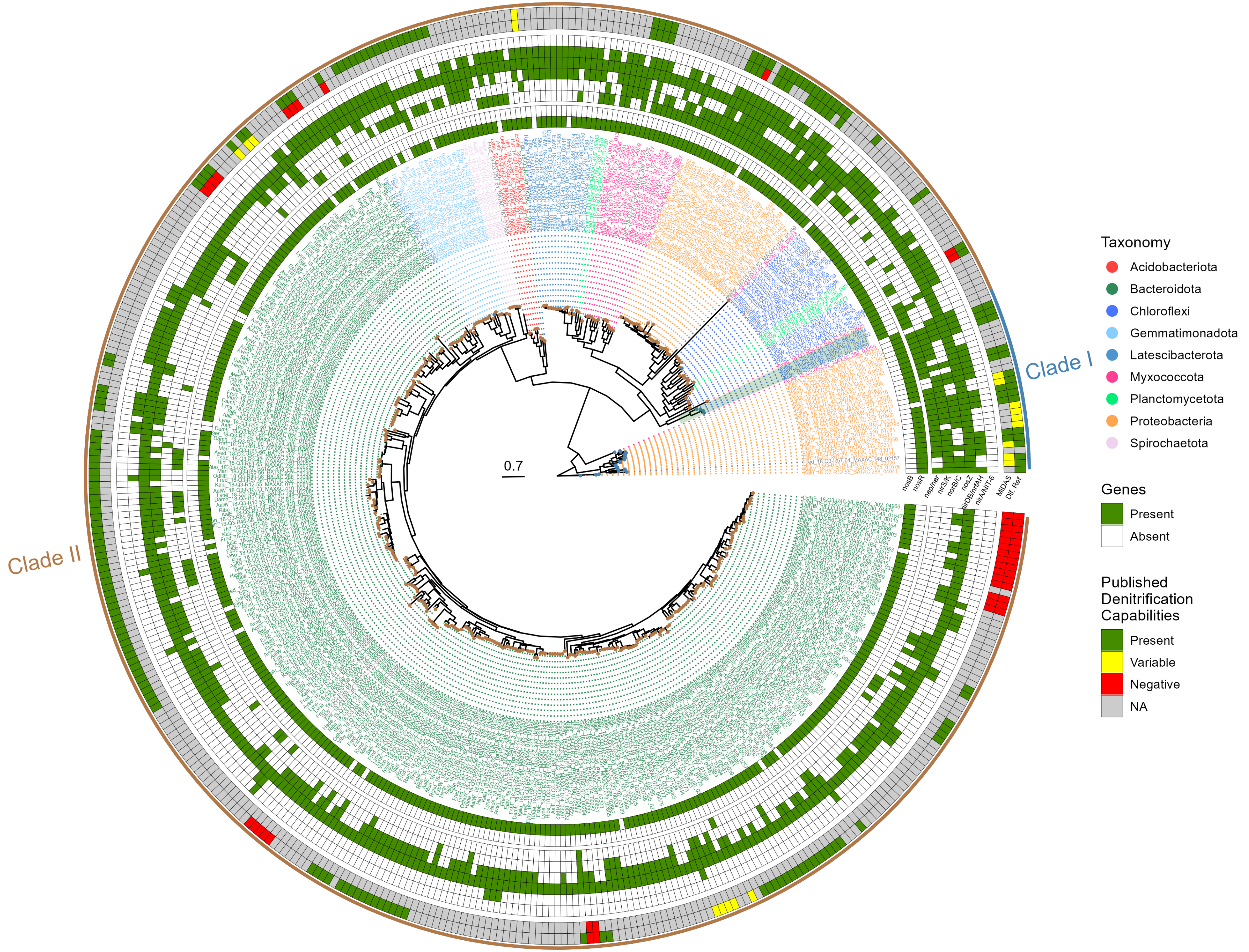

Figure S2: Circular maximum likelihood taxonomic tree of all clade annotated nosZ sequences on phylum level longer than 350aa (n=443) identified using the custom pipeline (see section 3.1). Each branch's tip is coloured by the respective tat (blue) or sec (brown) secretory pathway. The tip label, coloured by phylum, indicates the nosZ gene found in each HQ MAG, joined with a presence/absence indication of their respective accessory nosB/R, denitrifying and DNRA/ANR genes, along with annotated denitrifying capabilities from the MiDAS database and other references. The tree is constructed with ultrafast bootstrap values = 1000 and a scale bar denoting amino acid substitution rate. The taxonomic classification is made through the MiDAS 4.8.1 database (42).

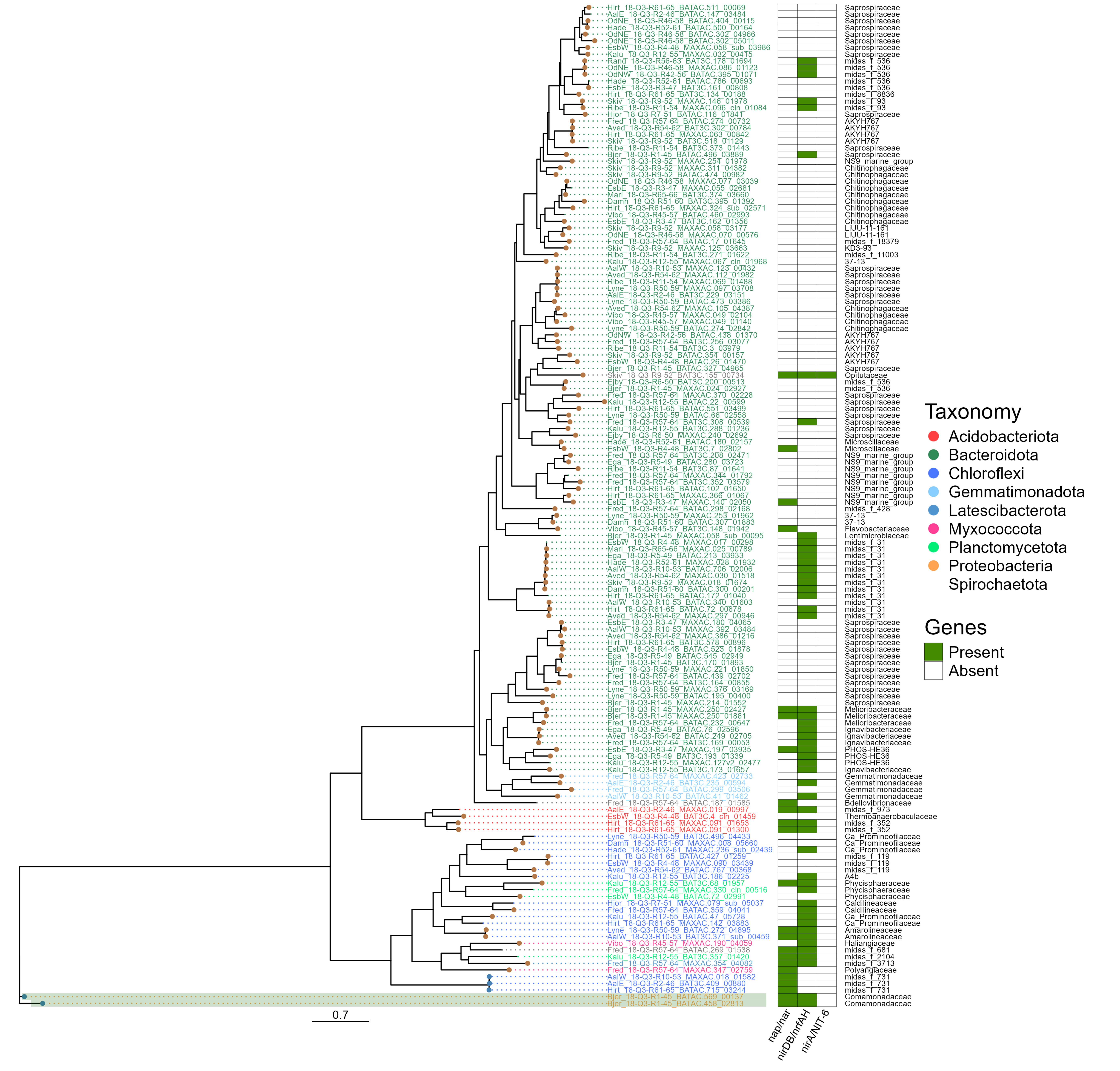

Figure S3: Maximum likelihood taxonomic tree of all non-denitrifying N2O-reducing HQ MAGs annotated on phylum level longer than 350aa (n=146) identified using the custom pipeline (see section 3.1). Illustrated as a phylogenetic tree with sec (brown) or tat (blue) secretory pathway at the tip of the tree, the labels indicates the nosZ gene found in each HQ MAG, joined with a heatmap of their respective genes associated with reduction of NO3- to NO2-, NO2- to NH4+, and N2 fixation genes. The green highlight indicates the two MAGs affiliated to nosZ clade I. The tree is constructed with ultrafast bootstrap values = 1000 and a scale bar denoting amino acid substitution rate. The taxonomic classification is made through the MiDAS 4.8.1 database (42).

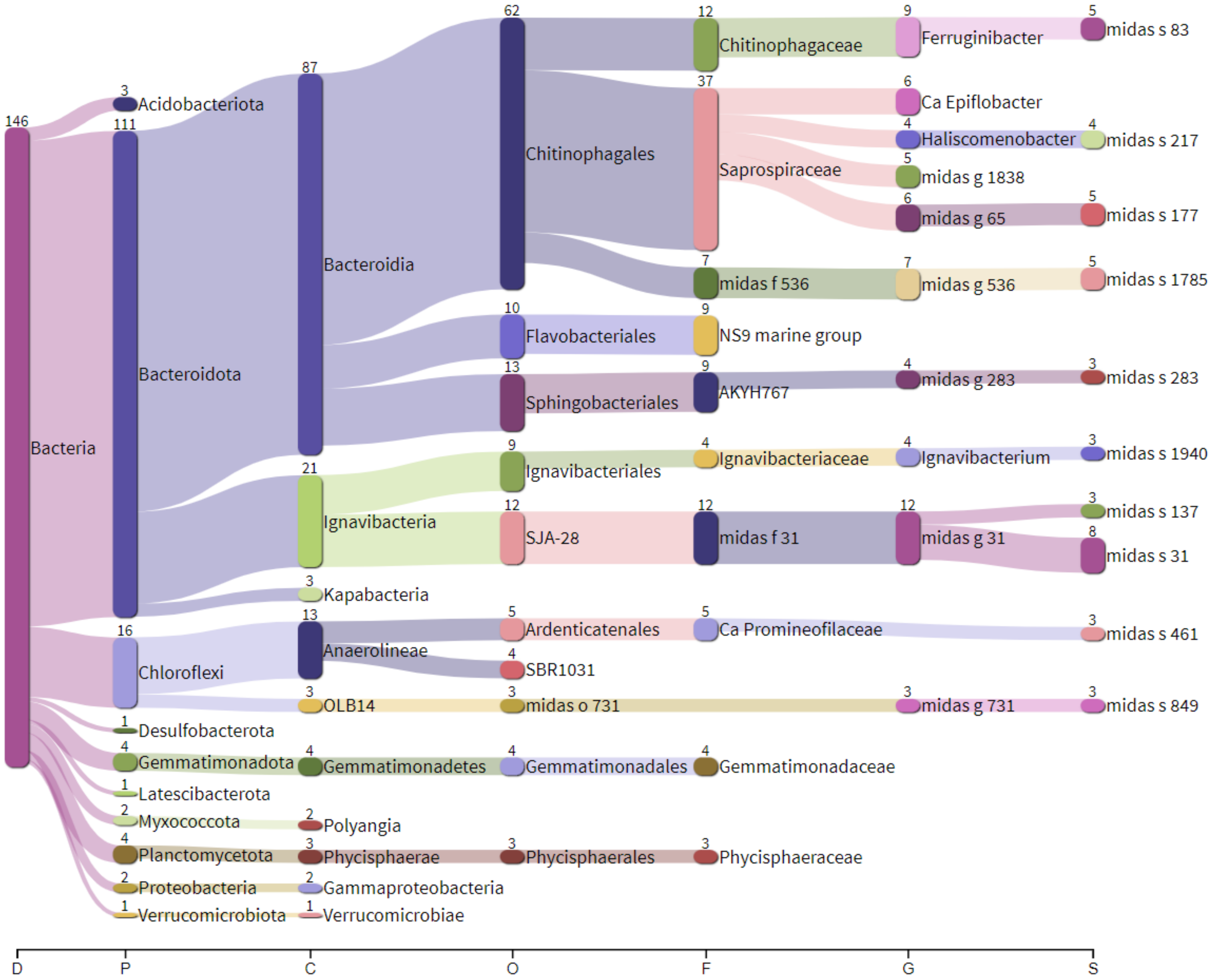

Figure S4: Sankey map of the top 10 taxa shown at each level of the non-denitrifying N_2O-reducers clade *nosZ* (n=146) containing HQ MAGs annotated with the MiDAS 4.8.1 database. Including MiDAS placeholders for annotations without binomial nomenclature.

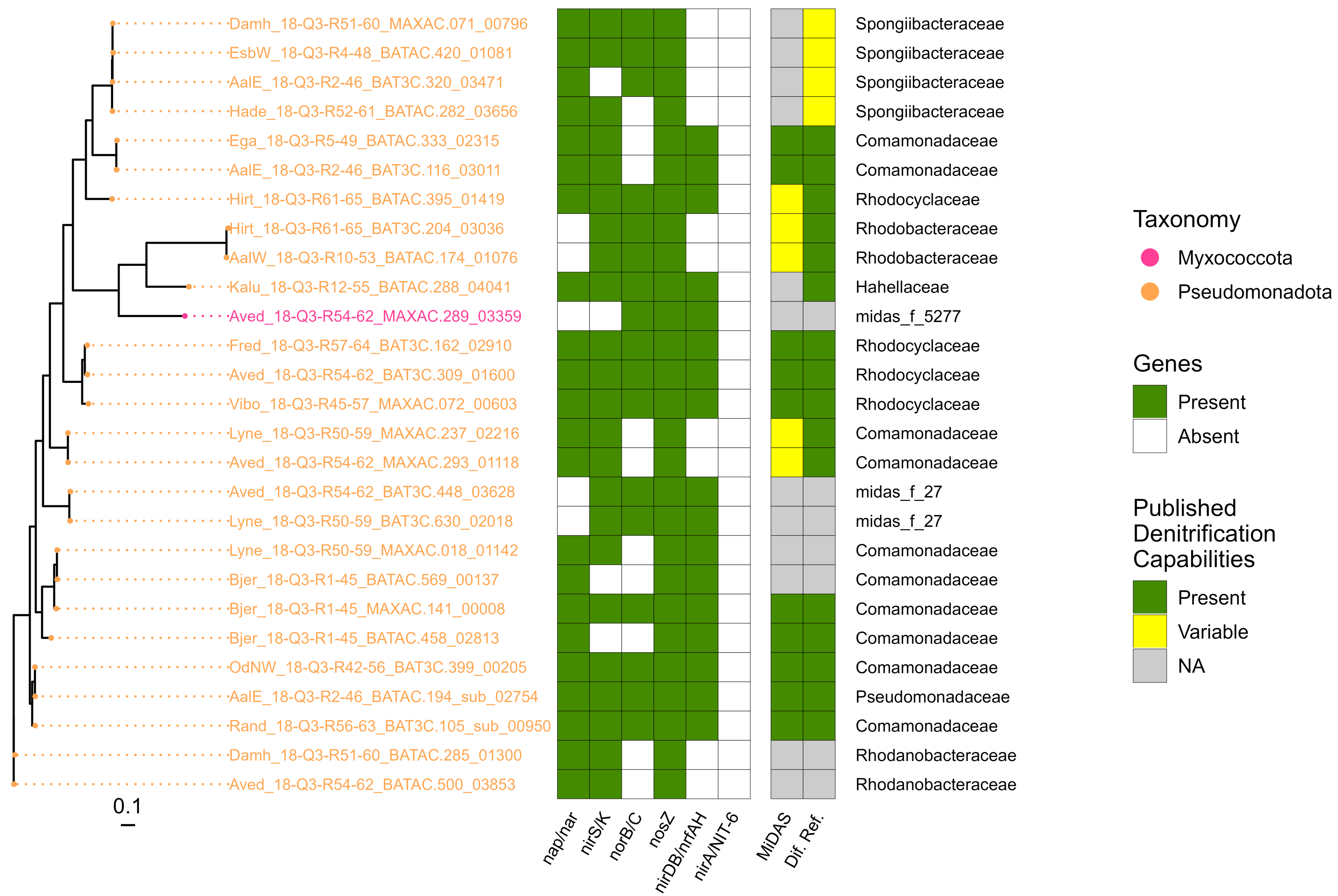

Figure S5: Maximum likelihood taxonomic tree of all clade I annotated nosZ sequences on phylum level longer than 350aa (n=33) identified using the custom pipeline (see section 3.1). Illustrated as a phylogenetic tree with sec (brown) or tat (blue) secretory pathway at the tip of the tree, the labels indicate the nosZ gene found in each HQ MAG, joined with a presence/absence indication of their respective denitrifying genes, along with annotated denitrifying capabilities from the MiDAS database and other references. The tree is constructed with ultrafast bootstrap values = 1000 and a scale bar denoting amino acid substitution rate. The taxonomic classification is made through the MiDAS 4.8.1 database (42).

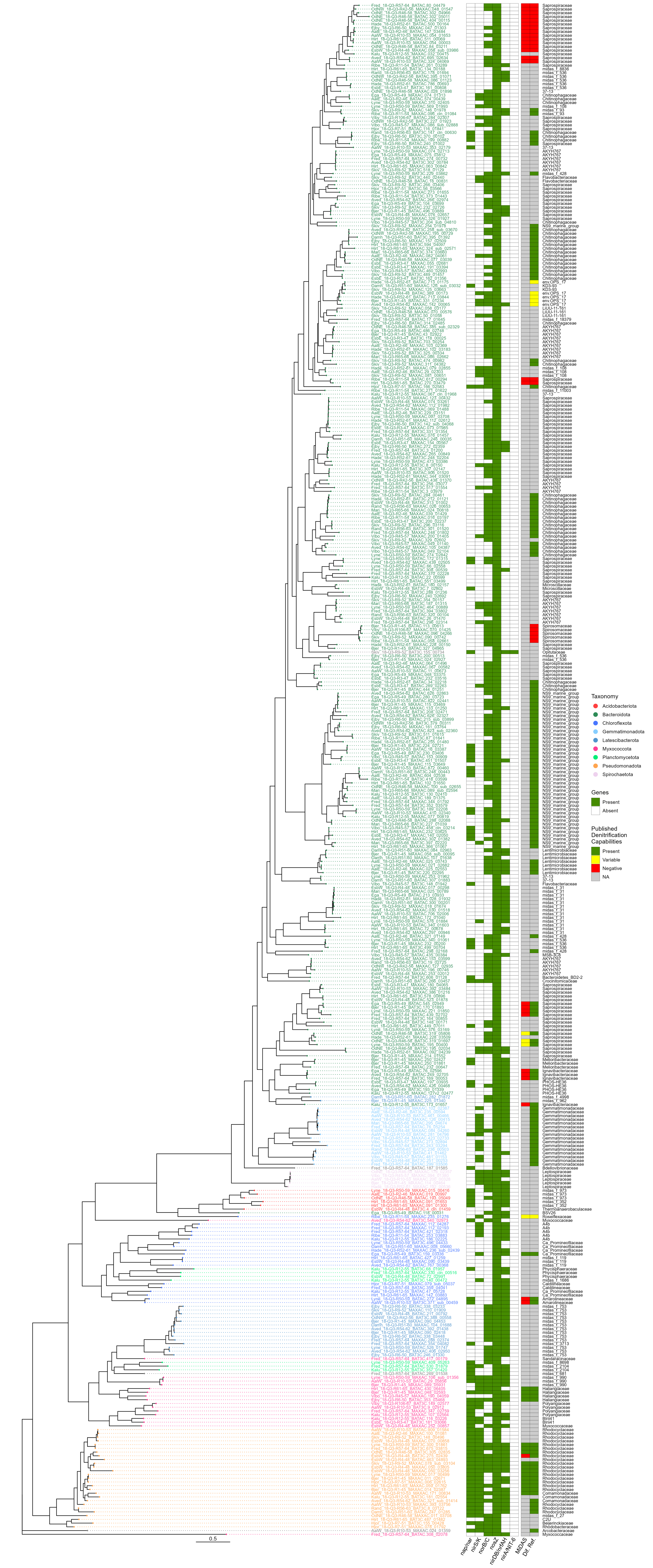

Figure S6: Maximum likelihood taxonomic tree of all clade I annotated nosZ sequences on phylum level longer than 350aa (n=395) identified using the custom pipeline (see section 3.1). Illustrated as a phylogenetic tree with sec (brown) or tat (blue) secretory pathway at the tip of the tree, the labels indicate the nosZ gene found in each HQ MAG, joined with a presence/absence indication of their respective denitrifying genes, along with annotated denitrifying capabilities from the MiDAS database and other references. The tree is constructed with ultrafast bootstrap values = 1000 and a scale bar denoting amino acid substitution rate. The taxonomic classification is made through the MiDAS 4.8.1 database (42).
