# Supplementary material for: Unraveling the genetic potential of nitrous oxide reduction in wastewater treatment: insights from metagenome-assembled genomes
Source: Appl Environ Microbiol. 2024 Aug 13;90(9):e02177-23. doi: 10.1128/aem.02177-23 (PMC11409646; doi:10.1128/aem.02177-23)
Supplement: Table S2 — List of custom-designed, clade-specific nosZ primers. [file aem.02177-23-s0004.docx]

Table S2: List of custom-designed primers clade-specific *nosZ* primers, including target taxonomy from the MiDAS 4.8.1 database (42).

| **Primer names** | **Taxonomic *nosZ* target group** |  | **Sequence 5’ to 3’** | **Bases** | **GC %** | **Tm** |
| --- | --- | --- | --- | --- | --- | --- |
| F1-CI | Chloroflexota | FWD | TGGGCAGGTGTTGGTGATCGGT | 22 | 59.1 | 63.4 |
| F2-CII | Chloroflexota | FWD | CATCGATCTGCGGGACTT | 18 | 55.6 | 54.7 |
| F3-CII | Chloroflexota | FWD | CTGAGTGAAACGAATGGCGA | 20 | 50.0 | 55.4 |
| F4-CII | Latescibacterota | FWD | GAAGCAGATCGTGCAGAG | 18 | 55.6 | 53.2 |
| F5-CII | Latescibacterota | FWD | GCCTAAACTTACCCGCCT | 18 | 55.6 | 54.7 |
| F6-CII | Myxococcota | FWD | GCGCATCCTCAAGTACAT | 18 | 50.0 | 52.4 |
| F7-CII | Proteobacteria | FWD | GTCGCCGCAAGTTCATCA | 18 | 55.6 | 55.9 |
| F8-CI | Proteobacteria | FWD | TTGCTTGGTGGGTTGGGG | 18 | 61.1 | 58.6 |
| F9-CI | Proteobacteria | FWD | GATGCGTGTTCCGGTGTT | 18 | 55.6 | 57.0 |
| F10-CI | Proteobacteria | FWD | TCTCGATTCCCCTGCCCA | 18 | 61.1 | 58.6 |
| R1-CII | Chloroflexota | REV | GGTTGATGTTGTAGAGGGGGA | 21 | 52.4 | 56.4 |
| R2-CII | Chloroflexota | REV | TGATCATCTGGGCGTAGT | 18 | 50.0 | 52.8 |
| R3-CII | Latescibacterota | REV | CATTGTCACGGGATTCAT | 18 | 44.4 | 49.8 |
| R4-CII | Latescibacterota | REV | TTGCACACCTTGCCCGGCT | 19 | 63.2 | 63.3 |
| R5-CII | Proteobacteria | REV | CGTACTTGGCGTTTTTGTC | 19 | 47.4 | 52.2 |
| R6-CI | Proteobacteria | REV | GATGATGGAGTCGTGCGG | 18 | 61.1 | 55.9 |
| R7-CI | Proteobacteria | REV | GGTTGGTCAGAATCAGGG | 18 | 55.6 | 52.7 |
| R8-CI | Proteobacteria | REV | CGTGGTTGGTGAGGATGATGG | 21 | 57.1 | 58.1 |
| R9-CII | Proteobacteria | REV | GTGGTCTCTTGGGGGTTG | 18 | 61.1 | 55.6 |
| R10-CII | Proteobacteria | REV | TGACTTCATCGCCCTTCT | 18 | 50.0 | 53.4 |
| R11-CII | Proteobacteria | REV | TACCGTCGAACCAGTCCA | 18 | 55.6 | 55.5 |
| F11-CII | Acidobacteriota | FWD | CCGCTGGCTCTTCGTCAA | 22 | 50.0 | 58.6 |
| F12-CII | Bacteroidota | FWD | TGTTTTTTCTGTTGACCCT | 19 | 36.8 | 49.5 |
| F13-CII | Bacteroidota | FWD | CCTCGTATTGCCCGTATT | 18 | 50.0 | 55.4 |
| F14-CII | Bacteroidota | FWD | ACCACTCYTCTCCTTTCC | 18 | 52.8 | 52.2 |
| F15-CII | Bacteroidota | FWD | AACCACTCCTCCCCTTTT | 18 | 50.0 | 53.4 |
| F16-CII | Bacteroidota | FWD | ACTCCTCBCCKTTYAYCA | 18 | 50.9 | 52.9 |
| F17-CII | Gemmatimonadetes | FWD | AAACGGGTACGGCTACAA | 18 | 45.0 | 55.9 |
| F18-CII | Gemmatimonadetes | FWD | CATGTCATCCCGGTCTTCT | 19 | 46.3 | 57.0 |
| F19-CII | Gemmatimonadetes | FWD | TCTTCACCACGTACAACTC | 18 | 43.9 | 58.6 |
| R12-CII | Acidobacteriota | REV | GCAGTAGTAGGGGAAGAC | 18 | 52.5 | 52.7 |
| R13-CII | Bacteroidota | REV | CACGTGTACCTTGTTGCCT | 19 | 52.6 | 59.4 |
| R14-CII | Bacteroidota | REV | CGCATATAACCCTGCATTTCCT | 22 | 45.5 | 55.3 |
| R15-CII | Bacteroidota | REV | CRCCCAYTTTCACACCTT | 18 | 50.0 | 53.0 |
| R16-CII | Bacteroidota | REV | ATCACCCATTTTCACACC | 18 | 44.4 | 50.0 |
| R17-CII | Bacteroidota | REV | GTGCTTGTTTTCCTCGAT | 18 | 44.4 | 50.3 |
| R18-CII | Gemmatimonadetes | REV | CGTACCCCTGCATCTCCT | 18 | 55.0 | 52.2 |
| R19-CII | Gemmatimonadetes | REV | CGGTGCAGTAGAAGGGGTAGA | 18 | 53.5 | 55.9 |
| R20-CII | Gemmatimonadetes | REV | CTGCTCGCCCTTGATCTT | 19 | 52.5 | 58.1 |
