# Supplementary material for: Unraveling the genetic potential of nitrous oxide reduction in wastewater treatment: insights from metagenome-assembled genomes
Source: Appl Environ Microbiol. 2024 Aug 13;90(9):e02177-23. doi: 10.1128/aem.02177-23 (PMC11409646; doi:10.1128/aem.02177-23)
Supplement: Table S3 — List of clade-specific nosZ-containing genera and their nitrogen-related metabolism. [file aem.02177-23-s0005.docx]

Table S3: List of clade-specific *nosZ*-containing genera and their nitrogen-related metabolism. The taxonomic classification is based on the MiDAS 4.8.1 database (1). The clade annotation is based on the custom pipeline (see section 3.1). Hits refer to number of HQ MAGs annotated within the genera in the examined 1083 HQ MAGs (2). Abbreviation from the MiDAS database IS: in situ NO_2_^-^ reduction, O: other NO_2_^-^ reduction, pos: positive, var: variable, neg: negative, na: not assessed.

| **Phylum** | **Class** | **Order** | **Family** | **Genus** | **clade** | **Hits** | **NO_2_^-^ IS** | **NO_2_^-^ O** | **Denitrifying reference** | **DNRA reference** |
| --- | --- | --- | --- | --- | --- | --- | --- | --- | --- | --- |
| *Bacteroidota* | *Bacteroidia* | *Chitinophagales* | *Saprospiraceae* | *Ca.* Epiflobacter | II | 17 | neg | na |  |  |
| *Bacteroidota* | *Bacteroidia* | *Flavobacteriales* | *Crocinitomicaceae* | *Crocinitomix* | II | 1 | na | na |  |  |
| *Bacteroidota* | *Bacteroidia* | *Chitinophagales* | *Chitinophagaceae* | *Ferruginibacter* | II | 20 | na | neg | (3)  Non-denitrifying N_2_O-reducer; (4) |  |
| *Bacteroidota* | *Bacteroidia* | *Flavobacteriales* | *Flavobacteriaceae* | *Flavobacterium* | II | 3 | na | na | (5, 6) |  |
| *Bacteroidota* | *Bacteroidia* | *Chitinophagales* | *Saprospiraceae* | *Haliscomenobacter* | II | 4 | neg | na | *nosZ* gene; (7, 8) |  |
| *Bacteroidota* | *Ignavibacteria* | *Ignavibacteriales* | *Ignavibacteriaceae* | *Ignavibacterium* | II | 4 | na | neg | *nosZ* gene; (9) | (10) |
| *Bacteroidota* | *Bacteroidia* | *Cytophagales* | *Spirosomaceae* | *Lacihabitans* | II | 5 | na | na |  |  |
| *Bacteroidota* | *Bacteroidia* | *Chitinophagales* | *Saprospiraceae* | *Lentimicrobium* | **II** | 4 | na | na | (11) |  |
| *Bacteroidota* | *Bacteroidia* | *Chitinophagales* | *Saprospiraceae* | *Lewinella* | **II** | 2 | na | na |  |  |
| *Bacteroidota* | *Bacteroidia* | *Chitinophagales* | *Saprospiraceae* | *Phaeodactylibacter* | **II** | 3 | na | var | (12) |  |
| *Bacteroidota* | *Bacteroidia* | *Chitinophagales* | *Chitinophagaceae* | *Terrimonas* | **II** | 19 | na | na | (12, 13) |  |
| *Campylobacterota* | *Campylobacteria* | *Campylobacterales* | *Arcobacteraceae* | *Arcobacter* | II | 1 | na | var | (14, 15) |  |
| *Chloroflexota* | *Anaerolineae* | C10-SB1A | *Amarolineaceae* | *Ca.* Amarolinea | II | 2 | na | neg | Non-denitrifying N_2_O reducer; (16) | (2, 16) |
| *Chloroflexota* | *Anaerolineae* | *Ardenticatenales* | *Ca_*Promineofilaceae | *Ca.* Promineofilum | II | 1 | na | pos | (17, 18) | (17) |
| *Chloroflexota* | *Chloroflexia* | *Chloroflexales* | *Roseiflexaceae* | *Kouleothrix* | II | 1 | neg | var | (19) |  |
| *Gemmatimonadota* | *Gemmatimonadetes* | *Gemmatimonadales* | *Gemmatimonadaceae* | *Gemmatimonas* | II | 1 | na | na | N_2_O reduction; (8) |  |
| *Myxococcota* | *Polyangia* | *Haliangiales* | *Haliangiaceae* | *Haliangium* | II | 4 | pos | na | (20, 21) |  |
| *Myxococcota* | *Polyangia* | *Polyangiales* | *Polyangiaceae* | *Pajaroellobacter* | II | 3 | na | na |  |  |
| *Myxococcota* | *Polyangia* | *Polyangiales* | *Polyangiaceae* | *Polyangium* | II | 1 | na | na |  |  |
| *Planctomycetota* | *Phycisphaerae* | *Phycisphaerales* | *Phycisphaeraceae* | *SM1A02* | II | 2 |  |  | (22) |  |
| *Pseudomonadota* | *Gammaproteobacteria* | *Xanthomonadales* | *Rhodanobacteraceae* | *Ahniella* | I | 2 | na | na |  |  |
| *Pseudomonadota* | *Gammaproteobacteria* | *Burkholderiales* | *Rhodocyclaceae* | *Ca.* Accumulibacter | I | 1 | var | pos | (23) |  |
| *Pseudomonadota* | *Gammaproteobacteria* | *Burkholderiales* | *Rhodocyclaceae* | *Dechloromonas* | II | 6 | pos | var | (21) |  |
| *Pseudomonadota* | *Gammaproteobacteria* | *Burkholderiales* | *Rhodocyclaceae* | *Denitratisoma* | II | 3 | na | pos | (24) |  |
| *Pseudomonadota* | *Gammaproteobacterial* | *Burkholderiales* | *Rhodocyclaceae* | *Ferribacterium* | II | 1 | na | var | (25) | (25) |
| *Pseudomonadota* | *Gammaproteobacteria* | *Pseudomonadales* | *Hahellaceae* | *Hahella* | I | 1 | na | na | (26) | (26) |
| *Pseudomonadota* | *Gammaproteobacteria* | *Burkholderiales* | *Comamonadaceae* | *Limnohabitans* | I | 2 | na | na | (27) |  |
| *Pseudomonadota* | *Gammaproteobacteria* | *Burkholderiales* | *Comamonadaceae* | *Ottowia* | I | 2 | na | var | (3) |  |
| *Pseudomonadota* | *Gammaproteobacteria* | *Pseudomonadales* | *Pseudomonadaceae* | *Pseudomonas* | I | 1 | na | pos | (28) |  |
| *Pseudomonadota* | *Alphaproteobacteria* | *Rhodobacterales* | *Rhodobacteraceae* | *Rhodobacter* | I | 2 | na | var | (29) |  |
| *Pseudomonadota* | *Gammaproteobacteria* | *Burkholderiales* | *Comamonadaceae* | *Rhodoferax* | I & II | 7 | pos | var | (21) |  |
| *Pseudomonadota* | *Gammaproteobacteria* | *Burkholderiales* | *Comamonadaceae* | *Sphaerotilus* | II | 1 | na | na | (30, 31) |  |
| *Pseudomonadota* | *Gammaproteobacteria* | *Burkholderiales* | *Rhodocyclaceae* | *Sulfuritalea* | II | 5 | pos | pos | (21) |  |
| *Pseudomonadota* | *Gammaproteobacteria* | *Burkholderiales* | *Rhodocyclaceae* | *Zoogloea* | O | 3 | pos | var | (32) |  |
| *Spirochaetota* | *Leptospirae* | *Leptospirales* | *Leptospiraceae* | *Leptospira* | II | 5 | na | na | nosZ gene; (33) |  |
| *Verrucomicrobiota* | *Verrucomicrobiae* | *Opitutales* | *Opitutaceae* | *Lacunisphaera* | II | 1 | na | na |  | (34) |

1. Dueholm MKD, Nierychlo M, Andersen KS, Rudkjøbing V, Knutsson S, Arriaga S, Bakke R, Boon N, Bux F, Christensson M, Chua ASM, Curtis TP, Cytryn E, Erijman L, Etchebehere C, Fatta-Kassinos D, Frigon D, Garcia-Chaves MC, Gu AZ, Horn H, Jenkins D, Kreuzinger N, Kumari S, Lanham A, Law Y, Leiknes T, Morgenroth E, Muszyński A, Petrovski S, Pijuan M, Pillai SB, Reis MAM, Rong Q, Rossetti S, Seviour R, Tooker N, Vainio P, van Loosdrecht M, Vikraman R, Wanner J, Weissbrodt D, Wen X, Zhang T, Nielsen PH, Albertsen M, Nielsen PH. 2022. MiDAS 4: A global catalogue of full-length 16S rRNA gene sequences and taxonomy for studies of bacterial communities in wastewater treatment plants. Nat Commun 13:1908.

2. Singleton CM, Petriglieri F, Kristensen JM, Kirkegaard RH, Michaelsen TY, Andersen MH, Kondrotaite Z, Karst SM, Dueholm MS, Nielsen PH, Albertsen M. 2021. Connecting structure to function with the recovery of over 1000 high-quality metagenome-assembled genomes from activated sludge using long-read sequencing. Nat Commun 12.

3. Xia Z, Wang Q, She Z, Gao M, Zhao Y, Guo L, Jin C. 2019. Nitrogen removal pathway and dynamics of microbial community with the increase of salinity in simultaneous nitrification and denitrification process. Science of the Total Environment 697.

4. Niu W, Guo J, Lian J, Ngo HH, Li H, Song Y, Li H, Yin P. 2018. Effect of fluctuating hydraulic retention time (HRT) on denitrification in the UASB reactors. Biochem Eng J 132:29–37.

5. Wang X, Ye C, Zhang Z, Guo Y, Yang R, Chen S. 2018. Effects of temperature shock on N2O emissions from denitrifying activated sludge and associated active bacteria. Bioresour Technol 249:605–611.

6. Zhou J heng, Yu H cheng, Ye K qiang, Wang H yu, Ruan Y jie, Yu J ming. 2019. Optimized aeration strategies for nitrogen removal efficiency: application of end gas recirculation aeration in the fixed bed biofilm reactor. Environmental Science and Pollution Research 26:28216–28227.

7. Jung J, Choi S, Jung H, Scow KM, Park W. 2013. Primers for amplification of nitrous oxide reductase genes associated with firmicutes and bacteroidetes in organic-compound-rich soils. Microbiology (United Kingdom) 159:307–315.

8. Park D, Kim H, Yoon S. 2017. Nitrous oxide reduction by an obligate aerobic bacterium, Gemmatimonas aurantiaca strain T-27. Appl Environ Microbiol 83.

9. Kim DD, Park D, Yoon H, Yun T, Song MJ, Yoon S. 2020. Quantification of nosZ genes and transcripts in activated sludge microbiomes with novel group-specific qPCR methods validated with metagenomic analyses. Water Res 185:116261.

10. Chen C, Han H, Meng Y, Gong H, Jia R, Xu T, Ding G-C, Li J. 2021. Total and denitrifying bacterial communities associated with the interception of nitrate leaching by carbon amendment in the subsoil. Appl Microbiol Biotechnol 105:2559–2572.

11. Wang H, Chen N, Feng C, Deng Y, Gao Y. 2020. Research on efficient denitrification system based on banana peel waste in sequencing batch reactors: Performance, microbial behavior and dissolved organic matter evolution. Chemosphere 253.

12. Ye J, Gao H, Domingo-Félez C, Wu J, Zhan M, Yu R, Smets BF. 2021. Insights into chronic zinc oxide nanoparticle stress responses of biological nitrogen removal system with nitrous oxide emission and its recovery potential. Bioresour Technol 327.

13. Zhang M, Gao J, Liu Q, Fan Y, Zhu C, Liu Y, He C, Wu J. 2021. Nitrite accumulation and microbial behavior by seeding denitrifying phosphorus removal sludge for partial denitrification (PD): The effect of COD/NO3− ratio. Bioresour Technol 323.

14. Callbeck CM, Pelzer C, Lavik G, Ferdelman TG, Graf JS, Vekeman B, Schunck H, Littmann S, Fuchs BM, Hach PF, Kalvelage T, Schmitz RA, Kuypers MMM. 2019. Arcobacter peruensissp. nov., a Chemolithoheterotroph Isolated from Sulfide- and Organic-Rich Coastal Waters off Peru. Appl Environ Microbiol 85.

15. Heylen K, Vanparys B, Wittebolle L, Verstraete W, Boon N, de Vos P. 2006. Cultivation of denitrifying bacteria: Optimization of isolation conditions and diversity study. Appl Environ Microbiol 72:2637–2643.

16. Andersen MH, McIlroy SJ, Nierychlo M, Nielsen PH, Albertsen M. 2019. Genomic insights into Candidatus Amarolinea aalborgensis gen. nov., sp. nov., associated with settleability problems in wastewater treatment plants. Syst Appl Microbiol 42:77–84.

17. McIlroy SJ, Karst SM, Nierychlo M, Dueholm MS, Albertsen M, Kirkegaard RH, Seviour RJ, Nielsen PH. 2016. Genomic and in situ investigations of the novel uncultured Chloroflexi associated with 0092 morphotype filamentous bulking in activated sludge. ISME Journal 10:2223–2234.

18. Xu X, Liu Y, Singh BP, Yang Q, Zhang Q, Wang H, Xia Z, Di H, Singh BK, Xu J, Li Y. 2020. NosZ clade II rather than clade I determine in situ N2O emissions with different fertilizer types under simulated climate change and its legacy. Soil Biol Biochem 150.

19. Kragelund C, Levantesi C, Borger A, Thelen K, Eikelboom D, Tandoi V, Kong Y, Van Der Waarde J, Krooneman J, Rossetti S, Thomsen TR, Nielsen PH. 2007. Identity, abundance and ecophysiology of filamentous Chloroflexi species present in activated sludge treatment plants. FEMS Microbiol Ecol 59:671–682.

20. Li L, Dong Y, Qian G, Hu X, Ye L. 2018. Performance and microbial community analysis of bio-electrocoagulation on simultaneous nitrification and denitrification in submerged membrane bioreactor at limited dissolved oxygen. Bioresour Technol 258:168–176.

21. McIlroy SJ, Starnawska A, Starnawski P, Saunders AM, Nierychlo M, Nielsen PH, Nielsen JL. 2016. Identification of active denitrifiers in full-scale nutrient removal wastewater treatment systems. Environ Microbiol 18:50–64.

22. Wu H, Cui M, Yang N, Liu Y, Wang X, Zhang L, Zhan G. 2022. Aerobic biocathodes with potential regulation for ammonia oxidation with concomitant cathodic oxygen reduction and their microbial communities. Bioelectrochemistry 144:107997.

23. Camejo PY, Oyserman BO, Mcmahon KD, Noguera DR. 2019. Integrated Omic Analyses Provide Evidence that a “Candidatus Accumulibacter phosphatis” Strain Performs Denitrification under Microaerobic Conditions.

24. Fahrbach M, Kuever J, Meinke R, Kämpfer P, Hollender J. 2006. Denitratisoma oestradiolicum gen. nov., sp. nov., a 17 β-oestradiol-degrading, denitrifying betaproteobacterium. Int J Syst Evol Microbiol 56:1547–1552.

25. Tan X, Yang YL, Liu YW, Li X, Zhu WB. 2021. Quantitative ecology associations between heterotrophic nitrification-aerobic denitrification, nitrogen-metabolism genes, and key bacteria in a tidal flow constructed wetland. Bioresour Technol 337.

26. Yuk S, Kamarisima, Azam AH, Miyanaga K, Tanji Y. 2020. The contribution of nitrate-reducing bacterium Marinobacter YB03 to biological souring and microbiologically influenced corrosion of carbon steel. Biochem Eng J 156.

27. Dang C, Liu S, Chen Q, Sun W, Zhong H, Hu J, Liang E, Ni J. 2021. Response of microbial nitrogen transformation processes to antibiotic stress in a drinking water reservoir. Science of the Total Environment 797.

28. Palleroni NJ. 2015. Pseudomonas , p. 1–1. *In* Bergey’s Manual of Systematics of Archaea and Bacteria. Wiley.

29. Imhoff JF. 2015. Rhodobacter, p. 1–12. *In* Bergey’s Manual of Systematics of Archaea and Bacteria. Wiley.

30. Deng S, Li D, Yang X, Zhu S, Li J. 2016. Process of nitrogen transformation and microbial community structure in the Fe(0)–carbon-based bio-carrier filled in biological aerated filter. Environmental Science and Pollution Research 23:6621–6630.

31. Pan Z, Guo T, Sheng J, Feng H, Yan A, Li J. 2021. Adding waste iron shavings in reactor to develop aerobic granular sludge and enhance removal of nitrogen and phosphorus. J Environ Chem Eng 9.

32. Thomson TR, Nielsen JL, Ramsing NB, Nielsen PH. 2004. Micromanipulation and further identification of FISH-labelled microcolonies of a dominant denitrifying bacterium in activated sludge. Environ Microbiol 6:470–479.

33. Yu K, Zhang T. 2012. Metagenomic and metatranscriptomic analysis of microbial community structure and gene expression of activated sludge. PLoS One 7.

34. Ruen-pham K, Graham LE, Satjarak A. 2021. Spatial variation of cladophora epiphytes in the Nan River, Thailand. Plants 10.
